# Neuroprotective Effects of STAT3 Inhibitor on Hydrogen Peroxide-Induced Neuronal Cell Death via the ERK/CREB Signaling Pathway

**DOI:** 10.1007/s11064-024-04252-3

**Published:** 2024-12-09

**Authors:** Seul-Ki Kim, Yong-Jin Kwon, Eun-Bi Seo, Hyun-Seung Lee, Jie Ohn Sohn, Hyun Mu Shin, Sung Joon Kim, Sang-Kyu Ye

**Affiliations:** 1 Department of Pharmacology and Biomedical Sciences, Seoul National University College of Medicine, Seoul, 03080 Republic of Korea; 2 Department of Cosmetic Science, Kyungsung University, Busan, 48434 Republic of Korea; 3 Ischemic/Hypoxic Disease Institute, Seoul National University College of Medicine, Seoul, 03080 Republic of Korea; 4 Wide River Institute of Immunology, Seoul National University, Hongcheon, 25159 Republic of Korea; 5 Department of Physiology, Seoul National University College of Medicine, Seoul, 03080 Republic of Korea; 6 Biomedical Science Project (BK21PLUS), Seoul National University College of Medicine, Seoul, 03080 Republic of Korea; 7 Neuro-Immune Information Storage Network Research Center, Seoul National University College of Medicine, Seoul, 03080 Republic of Korea

**Keywords:** STAT3 inhibition, Oxidative stress, Neuroprotection, ERK/CREB signaling pathway, Neurodegenerative diseases, Immediate early genes

## Abstract

This study investigates the neuroprotective potential of STAT3 inhibition in reducing oxidative stress-induced neuronal damage and apoptosis, a major factor contributing to the onset and progression of neurodegenerative diseases, including Alzheimer’s disease (AD). Our findings demonstrate that STAT3 inhibitors significantly enhance cell survival and reduce apoptosis in SH-SY5Y cells exposed to hydrogen peroxide. These protective effects are mediated through the ERK/CREB signaling pathway rather than direct suppression of STAT3 phosphorylation. Further analysis revealed that the ERK pathway is a critical mediator of CREB activation following STAT3 inhibition. The protective effects of STAT3 inhibitors were significantly reduced in the presence of the ERK inhibitor PD98059, underscoring the importance of the ERK/CREB axis in neuroprotection. We observed that STAT3 inhibitors promote CREB phosphorylation, leading to the upregulation of immediate early genes such as c-Fos, c-Jun, Arc, Egr-1, NR4A1, and Homer1a, as well as BDNF. These genes are crucial for synaptic plasticity and long-term memory formation, suggesting that STAT3 inhibition may ameliorate cognitive impairments in neurodegenerative conditions. Our results highlight the potential of STAT3 inhibitors to counteract oxidative stress and enhance cognitive functions by modulating the ERK/CREB signaling pathway. These findings provide valuable insights into the molecular mechanisms of STAT3 inhibition and support its therapeutic potential for treating neurodegenerative diseases.

**Supplementary Information:**

The online version contains supplementary material available at 10.1007/s11064-024-04252-3.

## Introduction

Oxidative stress is a major contributing factor to the onset of various diseases, including cancer, cardiovascular diseases, atherosclerosis, rheumatoid arthritis, chronic inflammatory conditions, ischemic/reperfusion injury, hypertension, diabetes, neurodegenerative diseases, and aging [1, 2]. Oxidative stress arises from an imbalance between the generation of reactive oxygen species (ROS) and the capacity of the antioxidant defense system to eliminate them [3]. At low or moderate concentrations, ROS exhibit beneficial effects, including defense against pathogens and physiological roles in cellular responses to oxygen deprivation [4]. However, excessive ROS production can lead to damage in various cellular structures and components, such as cell membranes, lipids, proteins, lipoproteins, and DNA. When the cell's ability to repair damage is exceeded, it may result in functional loss or eventually lead to cell death [5, 6]. The brain, due to its high oxygen consumption and increased generation of ROS, is prone to exposure to oxidative stress. Additionally, with its high content of oxidation-sensitive polyunsaturated fatty acids, the brain is an organ more susceptible to oxidative damage [7, 8]. Therefore, cellular injury and cell death resulting from oxidative stress are considered key pathological features of neurodegenerative diseases [9, 10]. Additionally, oxidative stress exacerbates various pathological processes, including neuroinflammation and synaptic dysfunction, further accelerating the progression of neurodegenerative diseases [11]. Furthermore, oxidative stress plays a pivotal role in tauopathies by promoting the hyperphosphorylation of tau proteins, leading to the formation of neurofibrillary tangles (NFTs), which contribute to neuronal dysfunction and degeneration [12]. Given its central role in these pathological mechanisms, targeting oxidative stress represents a promising therapeutic strategy to slow the progression of Alzheimer's disease (AD) and related neurodegenerative disorders [11].

AD is the most common neurodegenerative disorder marked by a deterioration in cognitive functions, including behavioral disturbances, memory impairment, and a decline in active thinking [13, 14]. The principal neuropathological features of AD involve the abnormal accumulation of amyloid leading to plaque formation, hyperphosphorylation of tau proteins resulting in the formation of neurofibrillary tangles within neuronal cells, synaptic loss, and neuronal death [15, 16]. Memory impairment, especially the deficiency in long-term memory (LTM), is a prominent feature in various neurodegenerative disorders, including AD. These deficiencies primarily arise from synaptic dysfunction and insufficient integration of LTM, resulting in cognitive decline and memory deficits [17, 18].

Since cAMP-response element binding protein (CREB) is implicated in regulating various biological functions in the brain, including proliferation, neuronal differentiation, neural development, and cell survival, the activation of CREB plays a crucial role in maintaining and advancing brain function [19, 20]. The activation of CREB is intricately regulated by various protein kinases. Among them, phosphatidylinositol 3-kinase (PI3K)/protein kinase B (Akt) and extracellular signal-regulated kinase 1/2 (ERK1/2) are the most well-known upstream regulators, inducing Ser133 phosphorylation of CREB and thereby enhancing its transcriptional activity [21]. CREB regulates intracellular survival signals in various cell types, including neurons. CREB activation maintains cell survival and prevents apoptosis by promoting the expression of genes associated with survival while inhibiting those linked to cell death [22, 23]. Additionally, CREB activation enhances the expression of stress response genes, enabling cells to cope with stressful conditions and enhance their survival capabilities [24, 25]. Recent studies suggest that the ERK/CREB signaling pathway contributes to suppressing cell damage and reducing cell death induced by oxidative stress [26, 27]. Therefore, the activation of CREB is anticipated to be a crucial target for the treatment and prevention strategies of diseases associated with oxidative stress, including neurodegenerative brain disorders. Moreover, CREB is pivotal in synaptic plasticity, learning, memory formation, long-term potentiation (LTP), integration, and enhancement [28, 29]. Activated CREB promotes the expression of immediate-early genes (IEGs) such as c-Fos, early growth response gene-1 (Egr-1), and activity-regulated cytoskeleton-associated protein (Arc), as well as target genes like brain-derived neurotrophic factor (BDNF), thereby contributing to the formation, consolidation, and enhancement of synaptic plasticity and LTM [30, 31]. Thus, enhancing the activation of CREB to improve cognitive function in AD is a very promising approach [32].

It is well-established that IEGs within neuronal cells play a crucial role in regulating synaptic plasticity and the processes of learning and memory formation [31, 33, 34]. In particular, a notable reduction in the expression of specific IEGs has been observed not only in in vitro and in vivo experiments mimicking AD models but also in the brains of AD patients. These changes signify more than mere indicators of cognitive impairment; they suggest that the diminished expression of these IEGs may not only be a consequence but could actively contribute to the onset or progression of cognitive deficits [35, 36]. BDNF, the most widely distributed neurotrophin in the central nervous system, plays a pivotal role in synaptic plasticity and neuronal survival [37]. Reduced serum levels of BDNF have been observed in various neurological conditions, including neurodegenerative diseases such as AD, Huntington's disease (HD), and Parkinson's disease (PD), as well as mood disorders like depression and bipolar disorder [38]. In AD, low BDNF levels significantly contribute to cognitive deficits by regulating LTP and synaptic plasticity [39, 40]. BDNF is a key contributor to synaptic plasticity and is acknowledged as a crucial element in brain function related to memory and learning [41]. Higher BDNF expression can delay cognitive decline and impede the pathological progression of AD [42]. Augmenting BDNF expression in rodent and primate models of AD has demonstrated efficacy in alleviating neuronal cell death and improving learning and memory [43]. Therefore, directly supplementing BDNF or indirectly stimulating BDNF expression is being considered as an effective strategy for the treatment of AD, aiming to elevate BDNF levels [44].

The Janus kinase (JAK)/signal transducer and transcriptional activator 3 (STAT3) signaling pathway is implicated in neurodegenerative diseases, including AD. Considering the pivotal role STAT3 plays in the pathophysiology of these disorders, therapeutic strategies focused on STAT3 inhibition show considerable potential. In an animal model with systemic inflammation, the administration of a STAT3 inhibitor not only protects against microglial activation and neuroinflammation, characteristic of AD pathophysiology, but also reduces beta-site amyloid precursor protein-cleaving enzyme 1 (BACE1) protein levels [45]. In animal models, inhibition of the JAK/STAT3 signaling pathway reduces reactive astrocyte reactivity, diminishing amyloid plaques and microglial cell activation, ultimately preventing neuroinflammation [46]. Additionally, the loss of STAT3 in reactive astrocytes and systemic treatment with a STAT3 inhibitor contribute to the restoration of cerebral network function, providing protection against spatial memory and learning impairments and improving cognitive decline [47, 48]. Treatment with a specific STAT3 inhibitor has demonstrated a reduction in neuroinflammatory plaques, cerebral amyloid angiopathy, oxidative stress, and neuroinflammation in experimental animal models of AD, leading to improved cognitive function and a protective effect on neurovascular function [49]. Nevertheless, research on the effects of STAT3 inhibition on neuronal cells remains insufficient. Particularly, the influence and detailed mechanisms of STAT3 in synaptic plasticity and cognitive improvement are still unclear. Therefore, further research is needed to explore the potential therapeutic use of STAT3 inhibitors in AD.

This study investigates the neuroprotective potential of STAT3 inhibition in reducing oxidative stress-induced neuronal damage and apoptosis, key drivers in the onset and progression of neurodegenerative diseases like AD. Our findings demonstrate that STAT3 inhibitors effectively mitigate oxidative damage and apoptosis by activating CREB via the ERK signaling pathway, a crucial mechanism for reducing cell death. Blocking this pathway with an ERK inhibitor abolished the protective effects of STAT3 inhibition, underscoring the central role of the ERK/CREB signaling pathway. Additionally, CREB activation and the upregulation of its target genes, essential for synaptic plasticity and LTM, suggest that STAT3 inhibition may offer a promising therapeutic strategy for addressing memory deficits in neurodegenerative diseases. These results highlight the therapeutic potential of STAT3 inhibition in the treatment of AD and other neurodegenerative conditions.

## Materials and Methods

### Chemicals and Reagents

Hydrogen peroxide (H_2_O_2_, 30%), PD98059, and LY294002 were purchased from Sigma-Aldrich (St. Louis, MO, USA). MTT (3-(4,5-dimethylthiazol-2-yl)-2,5-diphenyltetrazolium bromide) reagent was purchased from Duchefa Biochemie (Haarlem, Netherlands). Anti-p^Y705^-STAT3, anti-STAT3, anti-p^T202/Y204^-p44/42 MAPK (Erk1/2), anti-p44/42 MAPK (Erk1/2), anti-p^S473^-Akt, anti-Akt, anti-p^S133^-CREB, anti-CREB, anti-c-Fos, anti-Caspase-3 and anti-Caspase-9 were purchased from Cell Signaling Technology (Danvers, MA, USA). Anti-c-Jun and anti-BDNF were purchased from ABclonal (Wuhan, China). Anti-PARP was purchased from Oncogene (Boston, MA, USA). Anti-α-Tubulin was purchased from Abbkine (Wuhan, China). STAT3 inhibitors, including Stattic purchased from Sigma-Aldrich, AG490, and Nifuroxazide obtained from Santa Cruz Biotechnology (Dallas, TX, USA), and ODZ10117, a novel STAT3 inhibitor synthesized in-house by our research group [50]. All other chemicals were obtained from Sigma-Aldrich.

### Cell Culture

The cells were cultured under standard conditions at 37 °C in a humidified atmosphere with 5% CO_2_. SH-SY5Y, a human neuroblastoma cell line, and HT22, a mouse hippocampal neuronal cell line, were procured from the Korean Cell Line Bank (Seoul, Korea) and the American Type Culture Collection (Rockville, MD, USA), respectively. Dulbecco's modified Eagle's medium (DMEM, Hyclone, Logan, UT), supplemented with 10% heat-inactivated fetal bovine serum (FBS, Hyclone) and 1% penicillin/streptomycin solution (Capricorn Scientific GmbH), served as the culture medium for the cell lines.

### Cell Viability Analysis

Cellular viability was determined using the MTT analysis. Following the completion of the experiment, the culture medium was aspirated, and MTT solution was added to each well. The plate was then incubated at 37 °C for 2 h. Subsequently, the medium containing the MTT solution was carefully removed, and formazan crystals were dissolved using dimethyl sulfoxide (DMSO). Absorbance readings at 570 nm were measured using a microplate reader (Tecan, Männedorf, Switzerland). Results were represented as a percentage relative to the control.

### IncuCyte Cytotox Green assay

The analysis of cellular cytotoxicity was conducted using IncuCyte Cytotox Green Dye (Essence BioScience, Ann Arbor, MI, USA), following the manufacturer's instructions. IncuCyte Cytotox Green Dye, a cyanine nucleic acid dye that stains dead cells, is a fluorescence-based cell toxicity reagent designed for real-time detection of cell toxicity, and its measurement principles and analysis methods have been previously described [51]. After treatment with the STAT3 inhibitor, the Cytotox Dye-containing medium was added to achieve a final concentration of 250 nM, simultaneous with H_2_O_2_ treatment. The changes in cellular toxicity resulting from this process were observed in real-time using the IncuCyte Live-Cell Imaging System (Sartorius, Göttingen Germany). Cells were monitored continuously for 24 h, with images captured at 1 h intervals to acquire data. The obtained data were analyzed using IncuCyte 2021A software (Sartorius, Göttingen Germany), and the algorithm quantified the green fluorescence as total green object area (µM^2^/Image).

In SH-SY5Y cells, cytotoxicity levels continued to increase throughout the entire 24-h observation period. However, in HT22 cells, the highest level of cytotoxicity was observed at 12 h, after which the signal began to decline. This reduction in fluorescence could be attributed to the loss of dead cells, which may have detached from the plate, resulting in a decreased signal. To avoid potential misinterpretation of the cytotoxicity data, we focused our analysis on the first 12 h of data in HT22 cells, where the cytotoxicity was actively increasing.

### Oxidative Stress Analysis

Oxidative stress was assessed using the CellROX™ Green Reagent (Thermo Fisher Scientific, Waltham, MA, USA), a cell-permeable fluorogenic probe designed to detect ROS in live cells. The experimental protocol followed the cytotoxicity assay described previously, with the addition of the CellROX™ Green Reagent at a final concentration of 5 μM. Real-time imaging was conducted using the IncuCyte Live-Cell Imaging System. Both SH-SY5Y and HT22 cells were monitored for 12 h, with images acquired at 1-h intervals. Peak oxidative stress, indicated by maximal green fluorescence, was detected at 4 h in both cell types, followed by a subsequent decline in fluorescence intensity.

Since ROS levels reached their maximum at 4 h, the oxidative stress analysis was concentrated on this time point to capture the peak response. Fluorescence data collected at the 4-h time point were used to quantify oxidative stress, expressed as total green object area (µM^2^/Image), and analyzed using IncuCyte 2021A software.

### Western Blotting

For protein expression analysis, extraction of total cellular proteins and preparation of protein loading samples were conducted as previously described [51]. Equal quantities of protein were resolved on 6–15% SDS-PAGE and subsequently transferred onto nitrocellulose blotting membranes (GE Healthcare Life Sciences, Chicago, IL, USA). Blots underwent blocking with 5% skim milk in TBS containing Tween 20 (TBS-T) for 1 h, followed by an overnight incubation with primary antibodies at 4 °C. On the subsequent day, blots were probed with horseradish peroxidase-conjugated secondary antibodies (Enzo Life Sciences, Farmingdale, NY, USA). Thorough washes with TBS-T were performed between each step, involving three washes for 10 min each. Detection of protein bands was accomplished using the ECL chemiluminescence kit (Biomax, Seoul, Korea). Quantitative analysis of band intensity was conducted using ImageJ software (NIH, Bethesda, MD, USA). The band intensity results are included in the supplementary data (Supplementary Figs. S3–S13).

### RNA Extraction and Quantitative Real-Time PCR

Total RNA was isolated from cell samples using the RNAiso Plus reagent (Takara, Shiga, Japan) following the manufacturer's instructions. For cDNA synthesis, 1 μg of total RNA was used in a 20 μL reaction, following the manufacturer's protocol of the ReverTra Ace qPCR RT Master Mix (TOYOBO, Osaka, Japan). RNA concentration and purity were assessed using a NanoDrop spectrophotometer (Thermo Fisher Scientific), with all samples showing OD260/280 ratios between 1.8 and 2.0, indicative of high-quality RNA. Quantitative real-time PCR was carried out using BlasTaq™ 2X qPCR MasterMix (Applied Biological Materials, Richmond, BC, Canada) on the CFX Connect Real-Time PCR Detection System (Bio-Rad, Hercules, CA, USA). The reaction included an initial denaturation step at 95 °C for 5 min, followed by 40 cycles of denaturation at 95 °C for 10 s, annealing at 60 °C for 10 s, and extension at 72 °C for 30 s. A melt curve analysis was performed to confirm amplification specificity, ensuring the absence of nonspecific products. Negative controls and no-template controls (NTC) were included in each experiment to ensure the accuracy of the results. The mRNA expression data were internally standardized by normalizing to the expression levels of *GAPDH*. Relative quantification of gene expression was performed using the 2^−ΔΔCt^ method, with the expression levels of each sample calculated relative to the control group. The primer sequences utilized for qPCR are detailed in Table 1.

**Table 1:** Oligonucleotide sequences for the quantitative RT-PCR

Gene name		Sequence (5'→3')
*c-Fos (human)*	Forward	TGCAGCCAAATGCCGCAAC
	Reverse	TCGGTGAGCTGCCAGGATG
*c-Jun (human)*	Forward	GTCCTTCTTCTCTTGCGTGG
	Reverse	GGAGACAAGTGGCAGAGTCC
*Arc (human)*	Forward	ACAACAGGTCTCAAGGTTCCC
	Reverse	AGCCGACTCCTCTCTGTAGC
*Egr-1 (human)*	Forward	GGTCAGTGGCCTAGTGAGC
	Reverse	GTGCCGCTGAGTAAATGGGA
*NR4A1 (human)*	Forward	CCAAGTACATCTGCCTGGCTA
	Reverse	GACAACTTCCTTCACCATGCC
*Homer1a (human)*	Forward	TTTGGTTGCTCGCTCCAC
	Reverse	TAAGGCTGCGGGTTCAAA
*GAPDH (human)*	Forward	CTGACTTCAACAGCGACACC
	Reverse	TAGCCAAATTCGTTGTCATACC

### Apoptosis Analysis

Apoptosis was evaluated using the Annexin V-FITC and Propidium Iodide (PI) detection kit (Becton Dickinson Bioscience, San Jose, CA) following the manufacturer’s guidelines. Briefly, cells were subjected to experimental treatments, washed twice with ice-cold PBS, and resuspended in Annexin V binding buffer. Annexin V-FITC and PI were then introduced to the cell suspension, followed by gentle vortexing, and incubation at room temperature in the dark for 15 min. Subsequent to incubation, binding buffer was added, and the samples were subjected to analysis using flow cytometry (BD LSRFortessa, Becton-Dickinson Biosciences, San Jose, CA, USA). Cells positively stained with Annexin V were identified as apoptotic cells.

### Statistical Analysis

The data were analyzed using Microsoft Excel 2016 software and GraphPad Prism 5 (GraphPad Software, Inc, La Jolla, CA, USA). The results were expressed as the mean ± SD from at least three independent experiments. Statistical significance was calculated using one-way ANOVA followed by Tukey’s post-hoc test. A *p*-value of < 0.05 was considered significant for all statistical analyses.

## Results

### STAT3 Inhibitor Mitigates H_2_O_2_-Induced Neuronal Cell Damage and Exerts Cytoprotective Effects

**Fig. 1:**
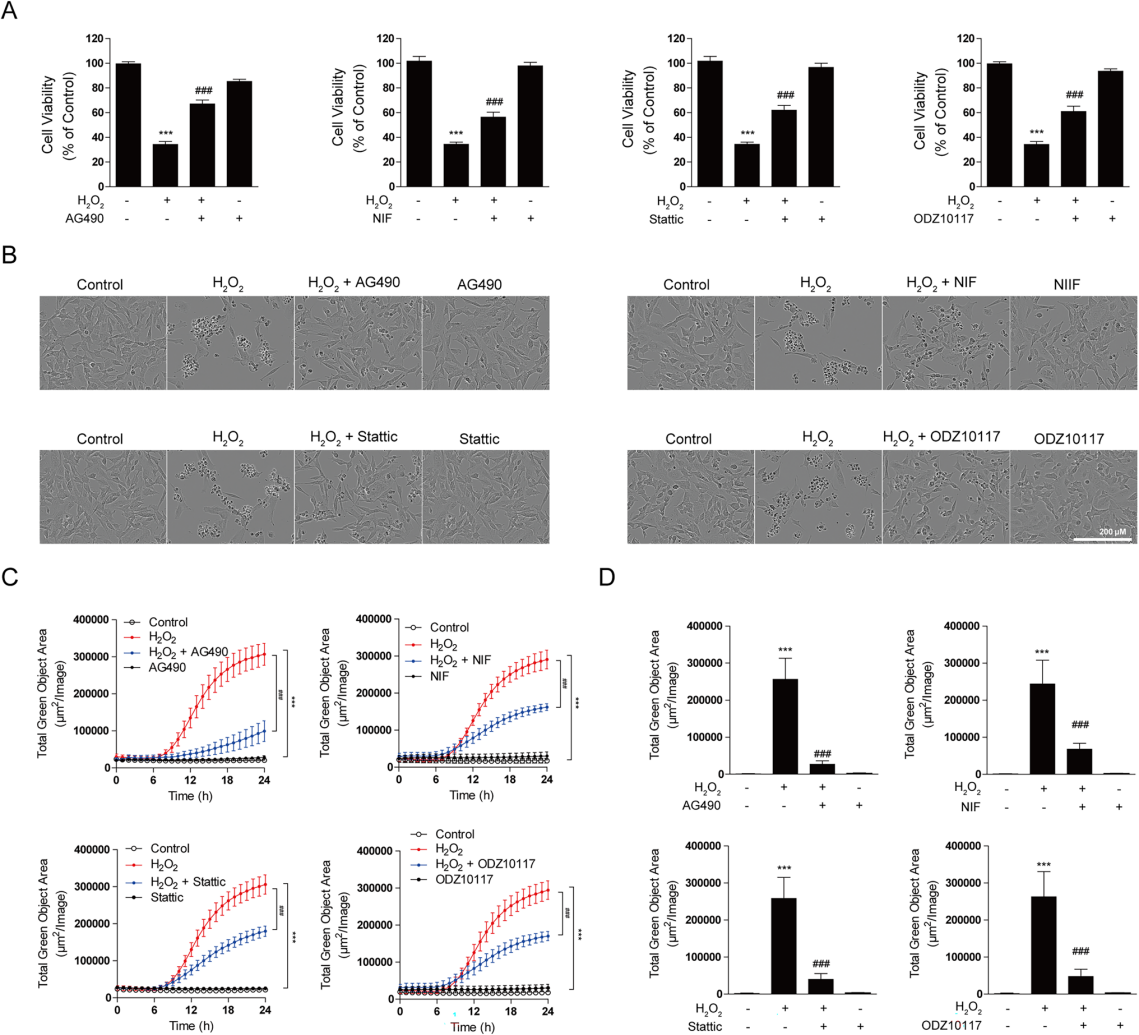
STAT3 inhibitor alleviates H_2_O_2_-induced oxidative damage and enhances cell viability. SH-SY5Y cells were pretreated with AG490 (75 µM), Nifuroxazide (10 µM), Stattic (1 µM), and ODZ10117 (10 µM) for 12 h, followed by exposure to 600 μM H_2_O_2_ for 24 h (**a**–**c**) or 4 h (**d**). **a** Cell viability was assessed using the MTT assay. **b** Morphological changes were observed through images captured using IncuCyte. **c** Cell death assessment was conducted through the quantification of deceased cells using the IncuCyte CytoTox Green Reagent. **d** Intracellular ROS levels were measured using the CellROX™ Green Reagent to assess oxidative stress at the 4-h time point during H_2_O_2_ exposure. The data are presented as the mean ± SD, representing a minimum of three independent experiments, with representative data shown. *p < 0.05, **p < 0.01, ***p < 0.005 compared with the control group. #*p* < 0.05, ##*p* < 0.01, ###*p* < 0.005 compared with H_2_O_2_-treated group. All statistical analyses were performed using one-way ANOVA followed by Tukey’s post-hoc test

Oxidative stress, a major contributor to neurodegenerative brain diseases, induces cell damage and apoptosis in neuronal cells [9, 52]. Thus, this study aimed to investigate whether a STAT3 inhibitor exerts protective effects against cell damage and death induced by H_2_O_2_ exposure. To determine the optimal concentration of H_2_O_2_ for inducing oxidative stress, we performed dose–response experiments in SH-SY5Y cells. Based on previous studies using the IC50 of H_2_O_2_ to induce oxidative damage in neuronal models [53, 54], we identified 600 μM H_2_O_2_ as the appropriate concentration, which resulted in approximately 50% cell viability (Supplementary Fig. 1a) and a significant increase in cytotoxicity (Supplementary Fig. 1b). This concentration was selected for subsequent experiments. To inhibit STAT3, we utilized a small-molecule inhibitor targeting STAT3 activation. This included the JAK2 inhibitors AG490 and Nifuroxazide, along with STAT3 selective inhibitors such as Stattic and ODZ10117. First, we examined whether the STAT3 inhibitor had a protective effect against the increased cell apoptosis induced by H_2_O_2_. SH-SY5Y cells were pretreated with the STAT3 inhibitor for 12 h, followed by exposure to H_2_O_2_ for an additional 12 or 24 h. The results demonstrated a significant reduction in cell survival with H_2_O_2_ treatment. However, cells pretreated with the STAT3 inhibitor before H_2_O_2_ exposure exhibited increased cell survival rates (Fig. 1a). Microscopic observations revealed that H_2_O_2_ treatment caused severe cell damage, resulting in cell detachment and a rounder cell morphology. However, these morphological changes were significantly inhibited by treatment with STAT3 inhibitor. Pretreatment with STAT3 inhibitor substantially alleviated H_2_O_2_-induced cell damage, resulting in a more stable cell appearance (Fig. 1b). Furthermore, H_2_O_2_ treatment increased cell toxicity, but the STAT3 inhibitor treatment considerably reduced this increase in cell toxicity (Fig. 1c). This indicates that the STAT3 inhibitor protects cells from cell damage caused by H_2_O_2_, thereby increasing cell survival rates. To validate the protective effects of the STAT3 inhibitor against oxidative stress, intracellular ROS levels were measured using CellROX™ Green Reagent as an indicator of oxidative damage. H_2_O_2_ exposure led to a significant elevation in ROS production in SH-SY5Y cells. However, pretreatment with the STAT3 inhibitor significantly reduced ROS levels, indicating that the inhibitor effectively mitigates oxidative stress (Fig. 1d). Collectively, these findings suggest that STAT3 inhibition exerts neuroprotective effects by alleviating oxidative stress and promoting cell survival in neuronal cells.

### STAT3 Inhibitor Attenuates H_2_O_2_-Induced Apoptotic Cell Death

**Fig. 2:**
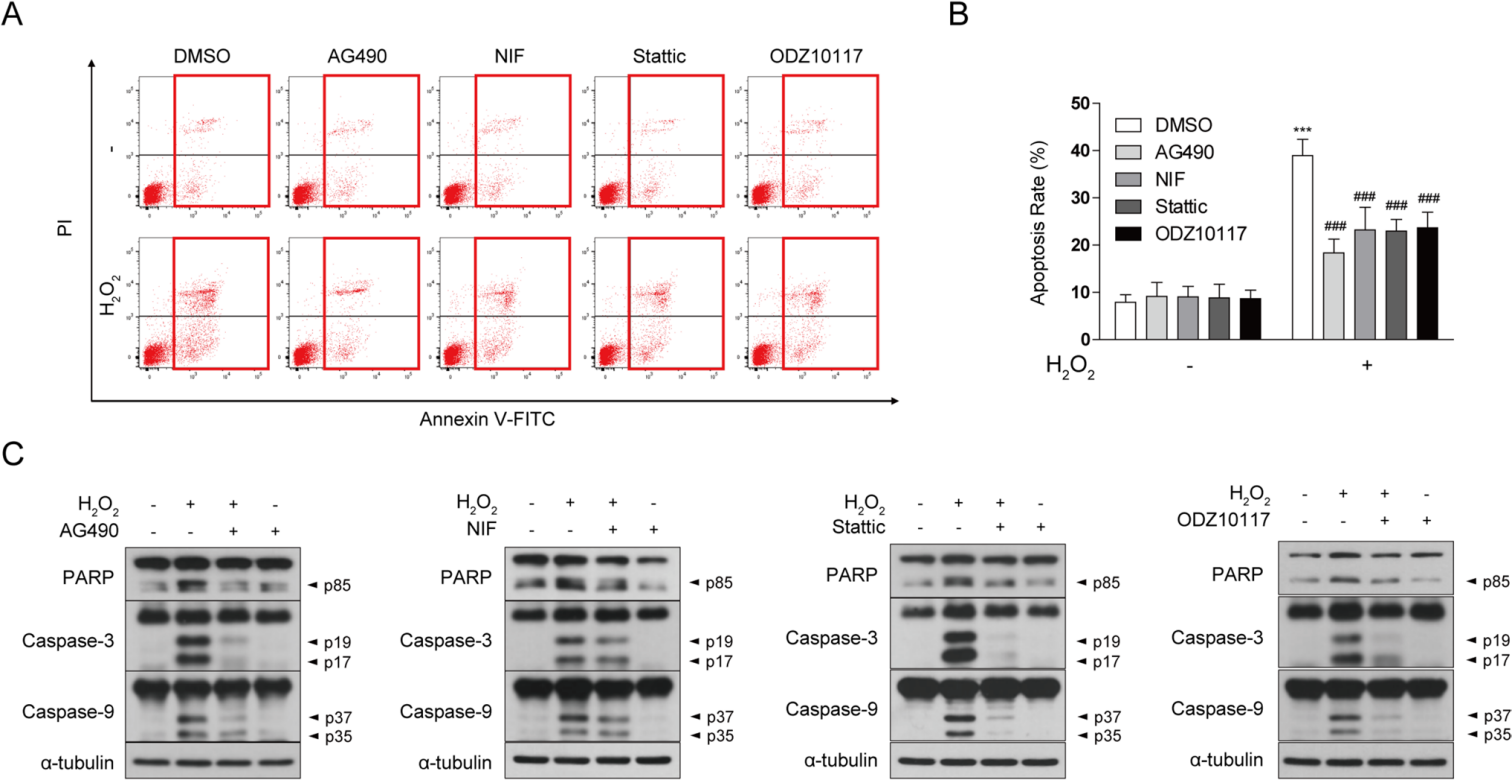
STAT3 inhibitor reduces H_2_O_2_-induced apoptosis and decreases caspase activation. SH-SY5Y cells were pretreated with AG490 (75 µM), Nifuroxazide (10 µM), Stattic (1 µM), and ODZ10117 (10 µM) for 12 h, followed by exposure to 600 μM H_2_O_2_ for 12 h. **a, b** Apoptotic cells were identified through Annexin V-FITC/PI staining, and flow cytometry analysis was conducted, considering Annexin V-positive cells as indicative of apoptosis. **c** Western blotting of cell lysates was conducted using the indicated antibodies, and α-tubulin was utilized as a loading control. The data are presented as the mean ± SD, representing a minimum of three independent experiments, with representative data shown. *p < 0.05, **p < 0.01, ***p < 0.005 compared with the control group. #*p* < 0.05, ##*p* < 0.01, ###*p* < 0.005 compared with H_2_O_2_-treated group. All statistical analyses were performed using one-way ANOVA followed by Tukey’s post-hoc test

Subsequently, we investigated whether the protective effects of STAT3 inhibition against H_2_O_2_-induced cellular damage were mediated through anti-apoptotic mechanisms. To assess this, we conducted Annexin V/PI staining and evaluated the expression of apoptosis-related proteins. In response to H_2_O_2_ treatment, the proportion of Annexin V-positive cells increased, but the presence of the STAT3 inhibitor significantly reduced the apoptotic cell rate (Fig. 2a, b). Additionally, H_2_O_2_ treatment increased caspase-dependent apoptosis, as indicated by a significant rise in caspase-3, caspase-9, and poly(ADP-ribose) polymerase (PARP) cleavage, which was alleviated by the STAT3 inhibitor treatment (Fig. 2c). In summary, this study demonstrates that a STAT3 inhibitor exerts protective effects against H_2_O_2_-induced cell damage and apoptosis. These findings highlight potential the potential of STAT3 inhibition as a therapeutic approach for oxidative stress-related neurodegenerative diseases and offer new insights into the protection of neuronal cells.

### STAT3 Inhibitor Induces CREB Activation Through The ERK Signaling Pathway

**Fig. 3:**
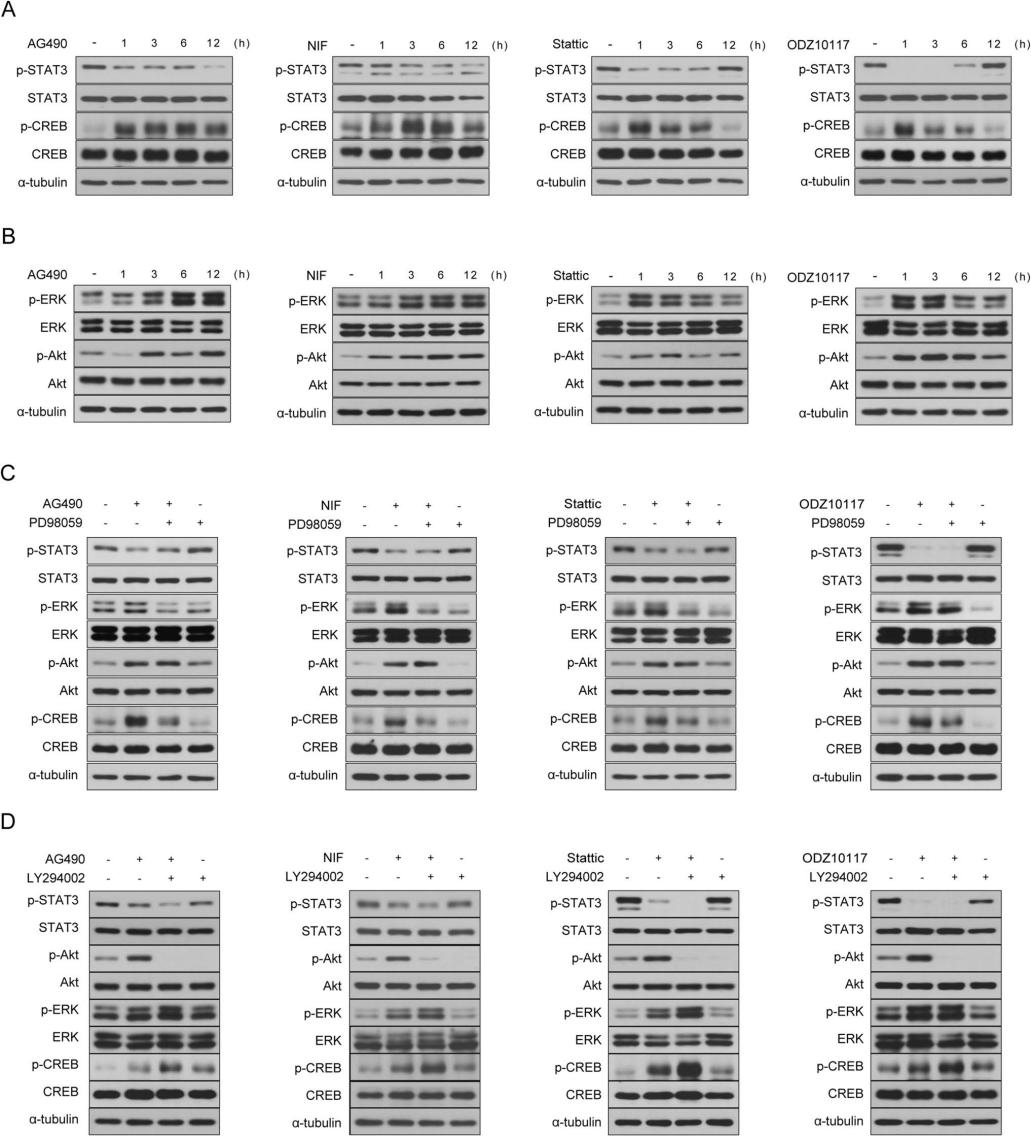
STAT3 Inhibitor induces CREB phosphorylation through the ERK signaling pathway. **a**, **b** SH-SY5Y cells were treated with AG490 (75 μM), Nifuroxazide (10 μM), Stattic (1 μM), and ODZ10117 (10 μM) for specified durations. **c** SH-SY5Y cells were pretreated with PD98059 (20 µM) for 30 min, followed by treatment with AG490 (75 µM), Nifuroxazide (10 µM), Stattic (1 µM), and ODZ10117 (10 µM) for 3 h. **d** SH-SY5Y cells were pretreated with Ly294002 (10 µM) for 30 min, followed by treatment with AG490 (75 µM), Nifuroxazide (10 µM), Stattic (1 µM), and ODZ10117 (10 µM) for 3 h. Western blotting of cell lysates was conducted using the indicated antibodies, and α-tubulin was utilized as a loading control

Next, we explored the fundamental mechanisms underpinning the protective effects of STAT3 inhibition on oxidative stress-induced neuronal cell death. Upon treating SH-SY5Y cells with H_2_O_2_, we did not observe an increase in the levels of phosphorylated STAT3 (Supplementary Fig. 2), which suggests that the protective actions of STAT3 inhibition are mediated through an alternative pathway, rather than through direct suppression of STAT3 activation. Given the established role of CREB in promoting neuronal growth and survival [19], coupled with the critical involvement of the ERK/CREB signaling pathway in enhancing cell survival and reducing apoptosis [26, 27], we hypothesized that CREB activation might serve as a pivotal mediator of the protective effects observed with STAT3 inhibition. Treatment with STAT3 inhibitors elicits rapid phosphorylation of CREB, resulting in a noticeable increase in p-CREB within 3 h of treatment. AG490 and Nifuroxazide induce upregulation of p-CREB, which persists from 3 to 12 h (Fig. 3a). Conversely, Stattic and ODZ10117 induce a peak in p-CREB levels at 1 h, followed by a gradual decline over time (Fig. 3a). The observed changes in p-CREB levels in response to STAT3 inhibitors closely correspond to the suppression patterns of p-STAT3, demonstrating a coherent link between the inhibition of STAT3 phosphorylation and subsequent regulation of CREB phosphorylation. The reduction of p-STAT3 by AG490 and Nifuroxazide gradually decreases until 12 h, whereas the downregulation of p-STAT3 by Stattic and ODZ10117 exhibits the most pronounced effect at 1 h, persisting until 6 h (Fig. 3a). In summary, these findings suggest that suppressing STAT3 phosphorylation influences CREB activation regulation. CREB phosphorylation is induced by upstream regulators in the ERK and Akt signaling pathways [21]. Additionally, prior studies have indicated that inhibiting STAT3 leads to feedback or compensatory activation, promoting the phosphorylation of ERK or Akt [55–59]. Thus, we hypothesized that the increased activation of CREB through STAT3 inhibition is mediated by the upstream regulators, specifically the ERK or Akt signaling pathways. Initially, we examined the effects of STAT3 inhibitors on ERK and Akt phosphorylation. Treatment with STAT3 inhibitors triggered the activation of ERK and Akt (Fig. 3b). Consistent with the observed changes in p-CREB following STAT3 inhibitor treatment, the levels of p-ERK and p-Akt induced by AG490 and Nifuroxazide gradually increased over time (Fig. 3b). In contrast, the changes in p-ERK and p-Akt induced by Stattic and ODZ10117 peaked at 1 or 3 h and gradually declined thereafter until 12 h (Fig. 3b). Following that, we investigated the involvement of the ERK or Akt signaling pathways in CREB activation mediated by STAT3 inhibition. To elucidate this, we assessed the phosphorylation status of CREB induced by STAT3 inhibitors in the presence or absence of the MEK/ERK inhibitor PD98059 or the PI3K/Akt inhibitor LY294002. The phosphorylation of CREB induced by STAT3 inhibitors was significantly diminished in the presence of PD98059 (Fig. 3c). In contrast, the co-treatment of STAT3 inhibitors and the Akt inhibitor led to a further enhancement in CREB phosphorylation compared to the group treated with STAT3 inhibitors alone (Fig. 3d). Furthermore, consistent with these findings, there was also a further enhancement observed in the phosphorylation of ERK (Fig. 3d). In summary, the ERK signaling pathway acts as an upstream regulator, mediating the activation of CREB in response to STAT3 inhibition.

### STAT3 Inhibitor Enhances the Expression of Genes Associated With Long-Term Memory Formation Through the ERK/CREB Signaling Pathway

**Fig. 4:**
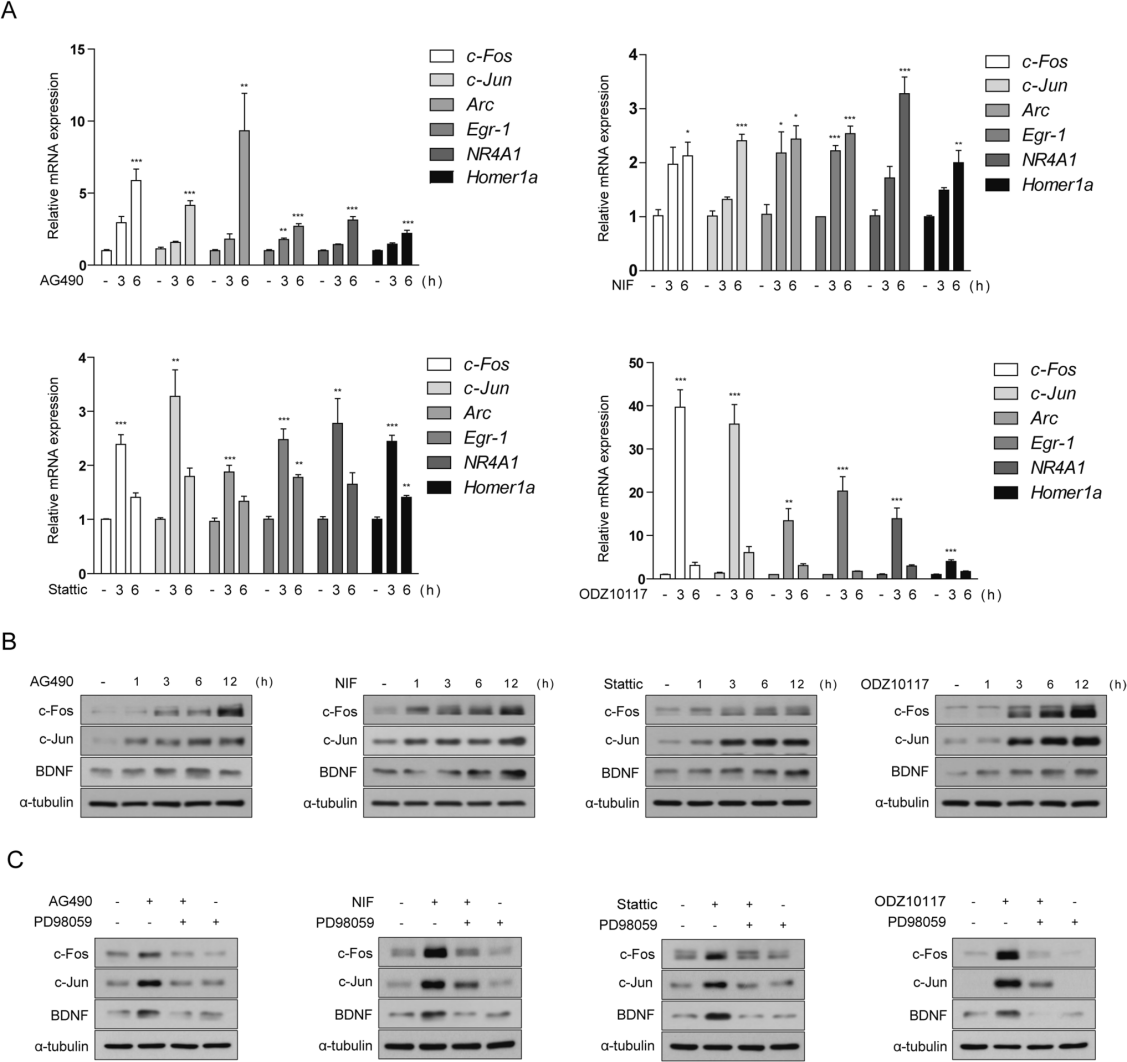
STAT3 inhibitor upregulate long-term memory-associated genes via the ERK/CREB signaling pathway. **a**, **b** SH-SY5Y cells underwent treatment with AG490 (75 µM), Nifuroxazide (10 µM), Stattic (1 µM), and ODZ10117 (10 µM) for the designated period. **a** Quantitative real-time PCR was employed to analyze mRNA levels of *c-Fos, c-Jun, Arc, Egr-1, NR4A1*, and *Homer1a*, with *GAPDH* serving as a loading control. **b** Western blotting of cell lysates was conducted using the indicated antibodies, and α-tubulin was utilized as a loading control. **c** SH-SY5Y cells were pretreated with PD98059 (20 µM) for 30 min, followed by subsequent treatment with AG490 (75 µM), Nifuroxazide (10 µM), Stattic (1 µM), and ODZ10117 (10 µM) for 12 h. Western blotting of cell lysates was conducted using the indicated antibodies, and α-tubulin was utilized as a loading control. The data are presented as the mean ± SD, representing a minimum of three independent experiments, with representative data shown. *p < 0.05, **p < 0.01, ***p < 0.005 compared with the control group. All statistical analyses were performed using one-way ANOVA followed by Tukey’s post-hoc test

Previous studies have suggested that inhibition of STAT3 may ameliorate cognitive impairment in AD [46–49], suggesting that STAT3 could represent a novel therapeutic target. However, further investigations are necessary to fully elucidate the mechanistic underpinnings of how STAT3 inhibition exerts its therapeutic effects on the disease. Dysfunction of synaptic function and impairment of LTM are closely associated with the cognitive decline observed in neurodegenerative brain disorders, including AD [17, 18, 60]. Upregulating genes related to synaptic plasticity and learning and memory has been proposed to protect against negative cognitive outcomes of neurodegeneration, including deficits in synaptic plasticity and learning/memory impairments. CREB positively regulates neuroplasticity, memory formation, enhancement, and learning in the adult brain [19, 20]. As CREB phosphorylation induces the transcription of memory-related genes, it serves as a key regulator in the pathways and mechanisms activated during synaptic strengthening and memory formation [28]. Based on our confirmation that STAT3 inhibitors induce CREB activation through the ERK signaling pathway, we aimed to investigate whether STAT3 inhibition regulates the expression of ERK/CREB-related IEGs in neuronal cells as a potential mechanism for ameliorating memory impairments. We scrutinized the mRNA levels of *c-Fos, c-Jun, Arc, Egr-1*, *NR4A1*, and *Homer1a* to assess their modulation in response to STAT3 inhibitor treatment. To evaluate the impact of STAT3 inhibitor treatment on IEGs expression, SH-SY5Y cells were exposed to STAT3 inhibitors for 3 and 6 h. By comparing with the untreated control, we observed a significant increase in mRNA levels of IEGs at both 3 and 6 h after STAT3 inhibitor treatment. The observed changes in mRNA levels of IEGs were consistent with the alterations observed in p-CREB expression (Fig. 4a). Specifically, AG490 and Nifuroxazide treatment elicited detectable mRNA levels of IEGs starting at 3 h, with a significant response observed at 6 h (Fig. 4a). Conversely, the effects induced by Stattic and ODZ10117 exhibited a more pronounced response at 3 h, followed by a subsequent decline at 6 h (Fig. 4a). Additionally, we examined the protein expression levels of the representative IEGs, c-Fos and c-Jun, as well as BDNF, a CREB target gene important for synaptic plasticity and LTM formation. SH-SY5Y cells were treated with STAT3 inhibitors in a time-dependent manner. We observed a significant increase in c-Fos, c-Jun, and BDNF protein levels starting at 6 h (Fig. 4b). Notably, the increased protein expression of c-Fos, c-Jun, and BDNF induced by STAT3 inhibition was not observed when cells were co-treated with PD98059 (Fig. 4c). Overall, our results suggest that STAT3 inhibition enhances ERK/CREB activation and promotes the expression of genes crucial for synaptic plasticity and LTM formation. These findings provide molecular insights into the therapeutic potential of STAT3 inhibition in neurodegenerative diseases, such as AD.

### STAT3 Inhibitor Reduces H_2_O_2_-Induced Cell Death by Modulating The ERK/CREB Signaling Pathway

**Fig. 5:**
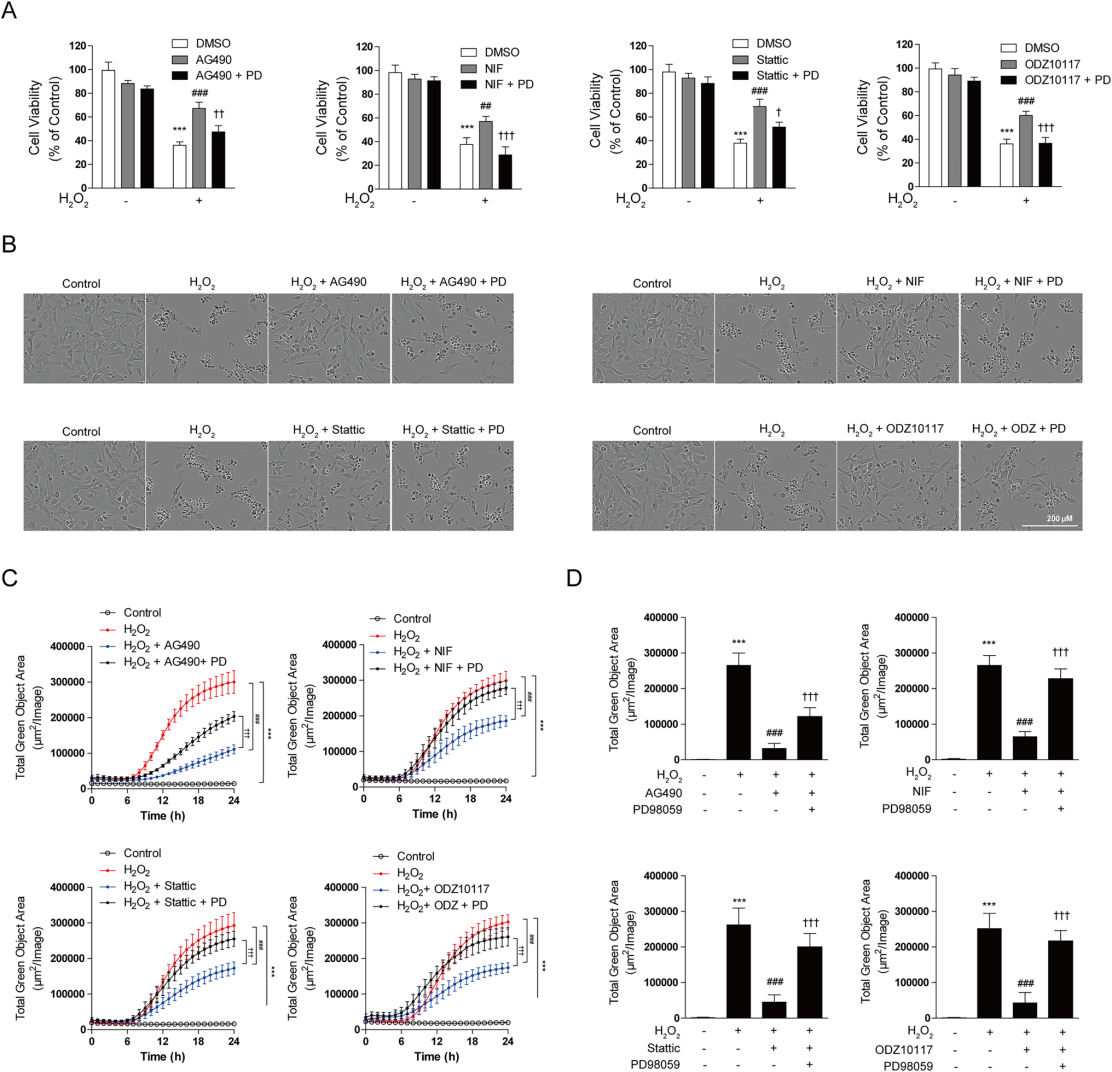
The neuroprotective effect of STAT3 inhibitor is mediated through the ERK/CREB signaling pathway. SH-SY5Y cells were pretreated with PD98059 (20 µM) for 30 min, followed by treatment with AG490 (75 µM), Nifuroxazide (10 µM), Stattic (1 µM), and ODZ10117 (10 µM) for 12 h. Subsequently, cells were exposed to 600 μM H_2_O_2_ for for 24 h (**a**–**c**) or 4 h (**d**). **a** Cell viability was assessed using the MTT assay. **b** Morphological changes were observed through images captured using IncuCyte. **c** Cell death assessment was conducted through the quantification of deceased cells using the IncuCyte CytoTox Green Reagent. **d** Intracellular ROS levels were measured using the CellROX™ Green Reagent to assess oxidative stress at the 4-h time point during H_2_O_2_ exposure. The data are presented as the mean ± SD, representing a minimum of three independent experiments, with representative data shown. *p < 0.05, **p < 0.01, ***p < 0.005 compared with the control group. #*p* < 0.05, ##*p* < 0.01, ###*p* < 0.005 compared with H_2_O_2_-treated group. †*p* < 0.05, ††*p* < 0.01, ††† *p* < 0.005 compared with H_2_O_2_ + STAT3 inhibitor-treated group. All statistical analyses were performed using one-way ANOVA followed by Tukey’s post-hoc test

Next, we investigated whether the protective effects of STAT3 inhibitor against cell damage and apoptosis induced by H_2_O_2_ are mediated through the ERK/CREB signaling pathway. SH-SY5Y cells were pretreated with PD98059 for 30 min, followed by treatment with STAT3 inhibitor for 12 h, and then exposed to H_2_O_2_ for 12 or 24 h. The results indicated that the presence of PD98059 significantly reduced the enhanced cell survival observed with the STAT3 inhibitor in response to H_2_O_2_-induced damage (Fig. 5a). Moreover, the protective effects of STAT3 inhibitor against cell loss and damage resulting from H_2_O_2_ exposure, as observed in morphological assessments, were negated when PD98059 was administered (Fig. 5b). This consistency was also confirmed in cell toxicity evaluations. In line with this, the mitigation of cell toxicity by the STAT3 inhibitor against H_2_O_2_ exposure was compromised by PD98059 (Fig. 5c). To investigate the role of the ERK/CREB signaling pathway in the STAT3 inhibitor's ability to reduce oxidative stress, we measured intracellular ROS levels in SH-SY5Y cells. H_2_O_2_ exposure significantly increased ROS production, but pretreatment with the STAT3 inhibitor substantially reduced these levels. However, this reduction in ROS was abolished in the presence of PD98059, suggesting that the ERK/CREB signaling pathway is critical for the STAT3 inhibitor's regulation of oxidative stress (Fig. 5d).

### STAT3 Inhibitor Suppresses H_2_O_2_-Induced Apoptosis via the ERK/CREB Signaling Pathway

**Fig. 6:**
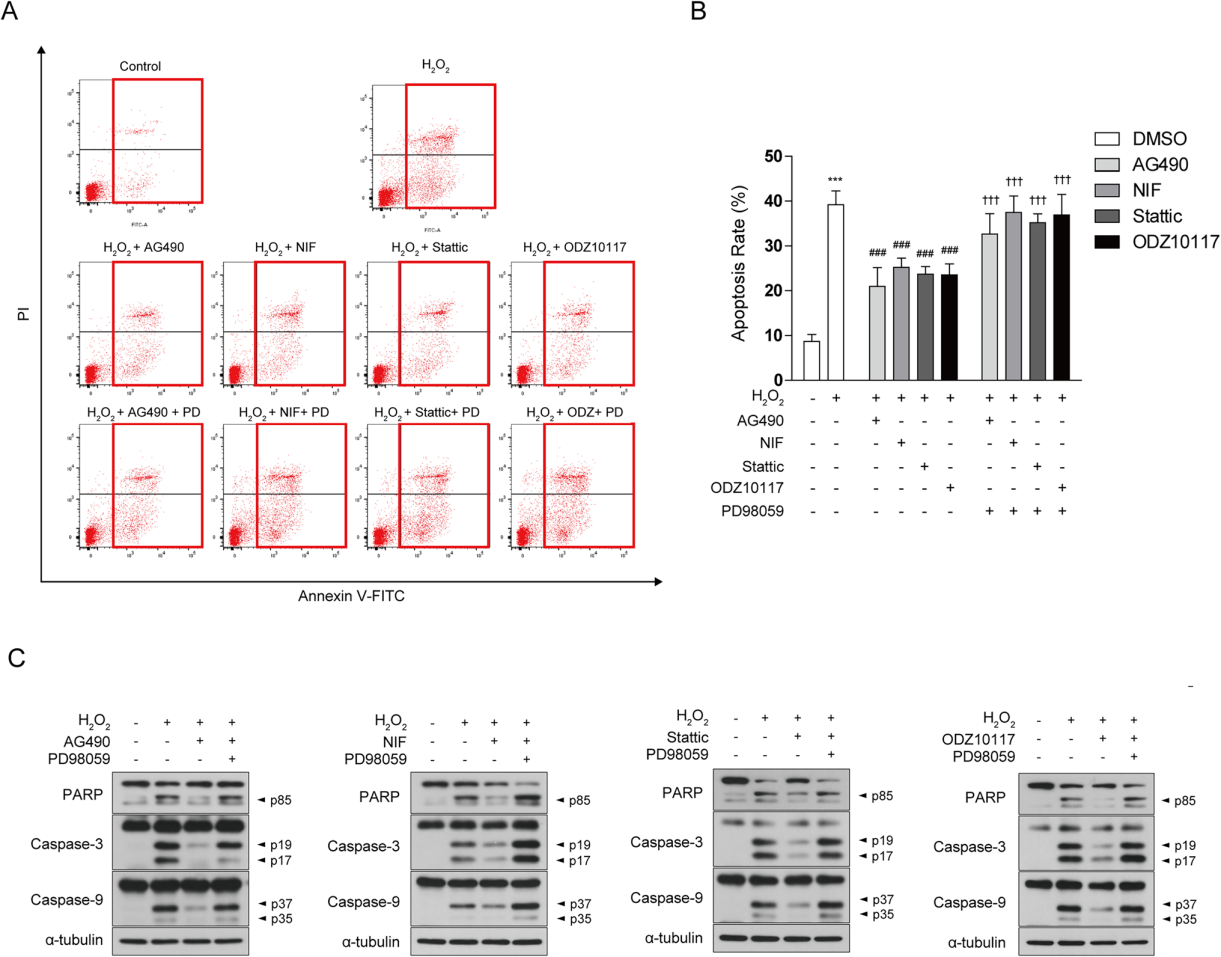
STAT3 inhibitor prevents H_2_O_2_-induced apoptosis through the ERK/CREB signaling pathway. SH-SY5Y cells were pretreated with PD98059 (20 µM) for 30 min, followed by treatment with AG490 (75 µM), Nifuroxazide (10 µM), Stattic (1 µM), and ODZ10117 (10 µM) for 12 h. Subsequently, cells were exposed to 600 μM H_2_O_2_ for for 12 h. **a, b** Apoptotic cells were identified through Annexin V-FITC/PI staining, and flow cytometry analysis was conducted, considering Annexin V-positive cells as indicative of apoptosis. **c** Western blotting of cell lysates was conducted using the indicated antibodies, and α-tubulin was utilized as a loading control. The data are presented as the mean ± SD, representing a minimum of three independent experiments, with representative data shown. *p < 0.05, **p < 0.01, ***p < 0.005 compared with the control group. #*p* < 0.05, ##*p* < 0.01, ###*p* < 0.005 compared with H_2_O_2_-treated group. †*p* < 0.05, ††*p* < 0.01, ††† *p* < 0.005 compared with H_2_O_2_ + STAT3 inhibitor-treated group. All statistical analyses were performed using one-way ANOVA followed by Tukey’s post-hoc test

Next, we explored whether the anti-apoptotic effects of the STAT3 inhibitor in H_2_O_2_-treated cells are mediated through the ERK/CREB signaling pathway. The results showed that the increased proportion of Annexin V-positive cells induced by H_2_O_2_ exposure was significantly inhibited by the STAT3 inhibitor. However, in the presence of PD98059, the protective effects of the STAT3 inhibitor were reversed (Fig. 6a, b). Additionally, similar results were observed in the evaluation of caspase-3, caspase-9, and PARP cleavage (Fig. 6c). In summary, these findings suggest that STAT3 inhibitor exerts protective effects against cell damage and apoptosis induced by H_2_O_2_, and these protective effects are associated with the ERK/CREB signaling pathway.

### STAT3 Inhibitor Activates the ERK/CREB Signaling Pathway in HT22 Cells

**Fig. 7:**
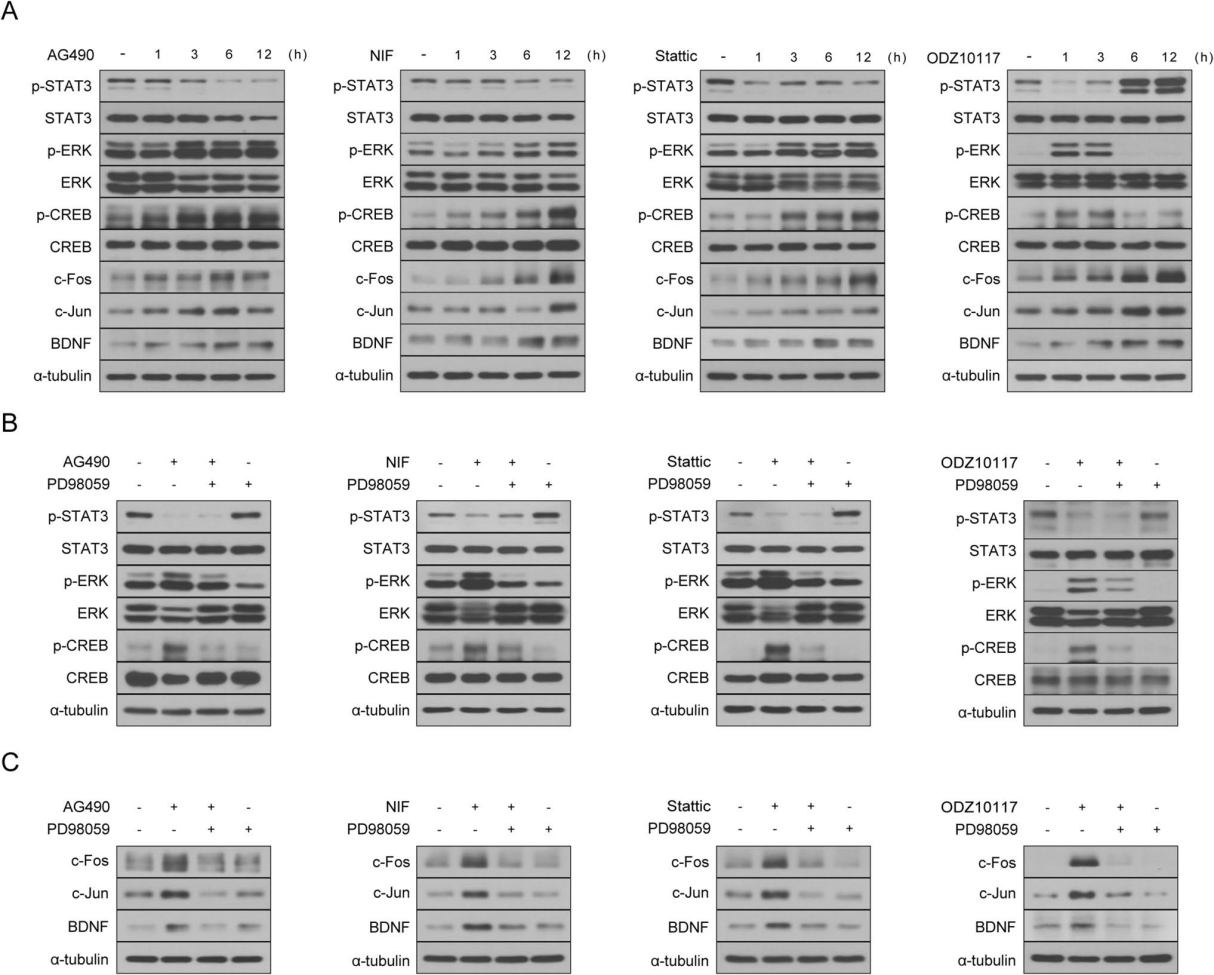
STAT3 inhibitor enhances the ERK/CREB signaling pathway and upregulates downstream targets in HT22 cells. **a** HT22 cells were treated with AG490 (75 μM), Nifuroxazide (10 μM), Stattic (1 μM), and ODZ10117 (30 μM) for specified durations. **b** HT22 cells were pretreated with PD98059 (20 μM) for 30 min, followed by exposure to AG490 (75 μM), Nifuroxazide (10 μM), Stattic (1 μM) for 6 h, or ODZ10117 (30 μM) for 3 h. **c** HT22 cells were pretreated with PD98059 (20 µM) for 30 min, followed by subsequent treatment with AG490 (75 µM), Nifuroxazide (10 µM), Stattic (1 µM), and ODZ10117 (30 µM) for a duration of 12 h. Western blotting of cell lysates was conducted using the indicated antibodies, and α-tubulin was utilized as a loading control

To further validate our findings, we conducted parallel experiments in the mouse hippocampal neuronal cell line, HT22, as an additional model to confirm the protective effects of STAT3 inhibition. Since the protective effects of STAT3 inhibition against cell death were mediated through the ERK/CREB signaling pathway in SH-SY5Y cells, we first examined whether STAT3 inhibition also activates ERK and CREB in HT22 cells. Additionally, we investigated whether STAT3 inhibition influences the expression of downstream target genes associated with the ERK/CREB signaling pathway. Consistent with our previous observations, treatment with the STAT3 inhibitor led to a marked increase in the phosphorylation of both ERK and CREB (Fig. 7a). Additionally, we observed a significant upregulation in the expression of the ERK/CREB signaling pathway downstream target genes c-Fos, c-Jun, and BDNF (Fig. 7a). Next, we investigated whether the increased activation of CREB, along with the upregulation of c-Fos, c-Jun, and BDNF, was dependent on ERK activation induced by STAT3 inhibition. Co-treatment with the ERK inhibitor significantly attenuated the STAT3 inhibitor-induced increases in p-ERK, p-CREB, as well as the expression of c-Fos, c-Jun, and BDNF (Fig. 7b, c). These results indicate that STAT3 inhibition promotes the activation of the ERK/CREB signaling pathway and the expression of its downstream target genes in HT22 cells, consistent with the findings in SH-SY5Y cells.

### STAT3 Inhibitor Confers Protection Against H_2_O_2_-Induced Cell Death in HT22 Cells Via the ERK/CREB Signaling Pathway

**Fig. 8:**
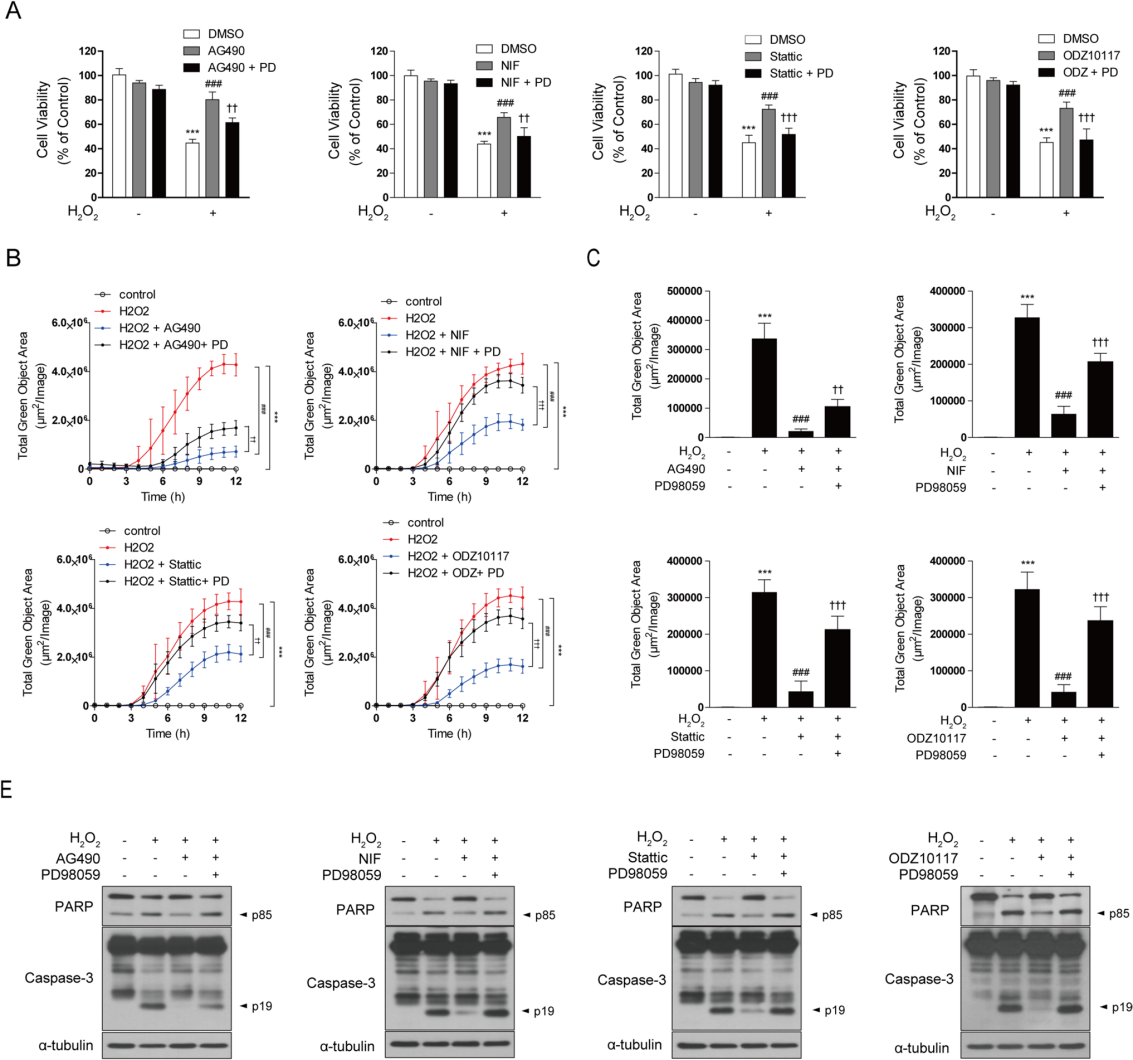
STAT3 inhibitor protects against H_2_O_2_-induced cell death via the ERK/CREB signaling pathway in HT22 cells. HT22 cells were pretreated with PD98059 (20 µM) for 30 min, followed by treatment with AG490 (75 µM), Nifuroxazide (10 µM), Stattic (1 µM), and ODZ10117 (10 µM) for 12 h. Subsequently, cells were then exposed to 600 μM H_2_O_2_ for varying durations: 24 h for (**a**, **b**), 4 h for (**c**), and 12 h for (**d**). **a** Cell viability was assessed using the MTT assay. **b** Cell death assessment was conducted through the quantification of deceased cells using the IncuCyte CytoTox Green Reagent. **c** Intracellular ROS levels were measured using the CellROX™ Green Reagent to assess oxidative stress at the 4-h time point during H_2_O_2_ exposure. **d** Western blotting of cell lysates was conducted using the indicated antibodies, and α-tubulin was utilized as a loading control. The data are presented as the mean ± SD, representing a minimum of three independent experiments, with representative data shown. *p < 0.05, **p < 0.01, ***p < 0.005 compared with the control group. #*p* < 0.05, ##*p* < 0.01, ###*p* < 0.005 compared with H_2_O_2_-treated group. †*p* < 0.05, ††*p* < 0.01, ††† *p* < 0.005 compared with H_2_O_2_ + STAT3 inhibitor-treated group. All statistical analyses were performed using one-way ANOVA followed by Tukey’s post-hoc test

Following the confirmation of the ERK/CREB signaling pathway activation by STAT3 inhibition in HT22 cells, we next aimed to determine whether the cytoprotective effects observed in SH-SY5Y cells under oxidative stress would also be present in HT22 cells. Given that STAT3 inhibition enhanced cell survival in SH-SY5Y cells through the ERK/CREB signaling pathway, we hypothesized that a similar mechanism would protect HT22 cells from H_2_O_2_-induced cell death. Consistent with the findings in SH-SY5Y cells, STAT3 inhibition significantly enhanced cell viability and reduced cytotoxicity in HT22 cells exposed to H_2_O_2_-induced oxidative stress (Fig. 8a, b). These protective effects were diminished upon co-treatment with the ERK inhibitor PD98059, confirming the involvement of the ERK/CREB signaling pathway. In addition, STAT3 inhibition lowered ROS levels elevated by H_2_O_2_, but this effect was also blocked by PD98059 (Fig. 8c), highlighting the critical role of the ERK/CREB signaling pathway in regulating oxidative stress. Moreover, STAT3 inhibition suppressed caspase-dependent apoptosis, as evidenced by decreased PARP and caspase-3 cleavage, a response that was similarly reversed by the addition of PD98059 (Fig. 8d). As in SH-SY5Y cells, the findings in HT22 cells highlight the importance of the ERK/CREB signaling pathway in mediating the cytoprotective effects of STAT3 inhibition against oxidative stress, reinforcing the potential of STAT3 inhibition as a therapeutic strategy in neuroprotection.

## Discussion

This study demonstrates the potential of STAT3 inhibition as a therapeutic strategy to mitigate neuronal damage and apoptosis caused by oxidative stress. Our key findings indicate that STAT3 inhibitors can effectively enhance cell survival and reduce apoptosis in SH-SY5Y cells exposed to H_2_O_2_. These protective effects are mediated through the regulation of the ERK/CREB signaling pathway, providing valuable insights into the molecular mechanisms underlying neuroprotection and laying the groundwork for therapies targeting neurodegenerative diseases such as AD. In neurodegenerative diseases, STAT3 has been primarily recognized for its role in cytokine signaling and inflammation, with less attention given to its involvement in neuronal function. Our study highlights STAT3 as a potential therapeutic target in neurodegenerative diseases and reveals novel aspects of STAT3 inhibition beyond conventional mechanisms.

Oxidative stress is well-established as a causative factor in the development of neurodegenerative diseases, including AD [9, 10]. Our results show that STAT3 inhibitors can reduce cell damage and death induced by H_2_O_2_. STAT3 inhibitors alleviate morphological changes and cell toxicity associated with oxidative stress, demonstrating their neuroprotective capabilities. These findings suggest that STAT3 inhibition effectively counters the detrimental effects of oxidative stress, making STAT3 inhibitors promising candidates for neuroprotection in conditions characterized by oxidative damage.

Importantly, our investigation into the mechanisms supporting the protective effects of STAT3 inhibition revealed that these effects are not mediated through the direct inhibition of STAT3 activation. Instead, we identified the ERK/CREB signaling pathway as a critical mediator. Phosphorylation of CREB, a transcription factor essential for neuronal survival and damage recovery, was significantly enhanced following STAT3 inhibitor treatment.

Our findings elucidate a molecular mechanism based on CREB activation through STAT3 inhibition and its relevance to the ERK and Akt signaling pathways. CREB activation is regulated by phosphorylation mediated by various upstream regulators, including ERK and Akt [21]. Previous studies suggested that STAT3 inhibition could trigger feedback or compensatory activation, leading to increased phosphorylation of ERK or Akt [55–59]. Our study corroborates this notion, showing that STAT3 inhibitors activate both ERK and Akt. However, only the ERK signaling pathway mediates CREB activation following STAT3 inhibition. Remarkably, simultaneous treatment with STAT3 and Akt inhibitors synergistically enhanced CREB phosphorylation, with concurrent augmentation in ERK phosphorylation and more robust inhibition of STAT3 phosphorylation compared to STAT3 treatment alone. These findings provide valuable insights into the intricate interactions among STAT3, ERK, Akt, and CREB, unraveling the underlying molecular mechanisms controlling CREB activation in response to STAT3 inhibition.

Beyond the context of oxidative stress, CREB activation has broad implications for the treatment of neurodegenerative diseases [20, 32]. CREB is pivotal in regulating genes associated with synaptic plasticity and memory formation, making it a crucial target for therapeutic interventions aimed at enhancing cognitive function. Previous studies have shown that enhancing CREB activity can improve cognitive deficits in animal models of neurodegenerative diseases [61], suggesting that STAT3 inhibitors might offer similar benefits. The regulation of IEGs such as c-Fos, c-Jun, Arc, Egr-1, NR4A1, and Homer1a by STAT3 inhibitors underscores the potential cognitive benefits of targeting STAT3. These genes are crucial for synaptic plasticity, learning, and memory, processes often impaired in neurodegenerative diseases. Upregulation of these IEGs, along with increased BDNF protein levels following STAT3 inhibition, suggests mechanisms by which cognitive decline could be mitigated under oxidative stress and neurodegenerative conditions. Previous studies have demonstrated that treatment with STAT3 inhibitors effectively improves cognitive impairment in animal models of AD [46–49]. Our research supports these findings by presenting new mechanisms that underline the dual role of STAT3 inhibitors in counteracting oxidative stress and enhancing cognitive functions impaired by neurodegenerative diseases.

In the broader context of therapeutic attempts for neurodegenerative disease treatment, CREB activation is considered an efficient and reliable target. The activation of the CREB signaling pathway induces the expression of numerous antioxidant genes, suggesting that CREB activation may represent an adaptive mechanism in response to oxidative damage. Furthermore, CREB plays a central role in pathways and mechanisms involved in synaptic enhancement and memory formation. However, concerns have been raised about the feasibility of implementing CREB-focused strategies due to the potential carcinogenicity of CREB. Nonetheless, emphasizing the restoration of impaired CREB phosphorylation observed in neurodegenerative diseases is crucial. Our study suggests that STAT3 inhibition may offer a novel therapeutic strategy for neurodegenerative brain diseases by inducing compensatory activation of ERK/CREB to balance cell survival. Notably, the well-known anticancer properties of STAT3 inhibitors could effectively address challenges associated with CREB-focused strategies.

Nevertheless, our research has several limitations that need to be considered. Firstly, our study heavily relied on in vitro experiments using small-molecule inhibitors. To validate the therapeutic potential of STAT3 inhibition in more physiologically relevant settings, additional in vivo studies using animal models and, ultimately, clinical trials will be necessary. Thoroughly elucidating the complex molecular mechanisms through which STAT3 inhibition activates the ERK/CREB signaling pathway and exploring the broad applicability of STAT3 inhibitors in protecting neuronal cell health in various neurodegenerative contexts requires additional investigation. Moreover, while our study focused on cell damage induced by H_2_O_2_, more research is needed to investigate the efficacy of STAT3 inhibitors in mitigating cell damage induced by other oxidative stress factors and explore potential benefits in various models of neurodegenerative diseases.

In summary, this study provides evidence that STAT3 inhibitors protect neuronal cells from oxidative stress-induced damage through the activation of the ERK/CREB signaling pathway. The enhancement of CREB phosphorylation and the upregulation of IEGs associated with synaptic plasticity and memory formation suggest that STAT3 inhibitors hold promise not only for mitigating cell death but also for supporting cognitive function in neurodegenerative diseases. These insights pave the way for developing targeted therapies that harness the protective potential of STAT3 inhibition, contributing to a better understanding and treatment of conditions characterized by oxidative stress and neuronal degeneration.

## Supplementary Information

Below is the link to the electronic supplementary material.

Figure S1. Determination of the optimal H_2_O_2_ concentration for inducing oxidative stress in SH-SY5Y cells. SH-SY5Y cells were treated with increasing concentrations of H_2_O_2_ (100–1000 μM) for 24 h. (A) Cell viability was assessed using the MTT assay. (B) Cell death assessment was conducted through the quantification of deceased cells using the IncuCyte CytoTox Green Reagent.
Supplementary file1 (TIF 25520 KB)

Figure S2. Effects of H_2_O_2_ on STAT3 phosphorylation in SH-SY5Y cells. (A) SH-SY5Y cells were treated with H_2_O_2_ (600 µM) for various time points. (B) SH-SY5Y cells were treated with different concentrations of H_2_O_2_ for 8 h. Western blotting of cell lysates was conducted using the indicated antibodies, and α-tubulin was utilized as a loading control. The data are presented as the mean ± SD, representing a minimum of three independent experiments, with representative data shown. *p < 0.05, **p < 0.01, ***p < 0.005 compared with the control group. All statistical analyses were performed using one-way ANOVA followed by Tukey’s post-hoc test.
Supplementary file2 (TIF 25518 KB)

Figures S3–S13. Quantitative analysis of Western blot band intensities from main and supplementary data. The band intensities from the Western blot analyses, including those from the main figures and supplementary materials, were quantified using ImageJ software and normalized to their respective controls. Relative protein expression levels are presented as bar graphs in each figure. The specific normalization controls for each graph are indicated within the corresponding figure. The data are presented as the mean ± SD, representing a minimum of three independent experiments, with representative data shown. NS: not significant, *p < 0.05, **p < 0.01, ***p < 0.005 compared with the control group. #*p* < 0.05, ##*p* < 0.01, ###*p* < 0.005 compared with H_2_O_2_-treated group. †*p *< 0.05, ††*p *< 0.01, ††† *p* < 0.005 compared with H_2_O_2_+ STAT3 inhibitor-treated group. All statistical analyses were performed using one-way ANOVA followed by Tukey’s post-hoc test.
Figures S3. Quantitative analysis of Western blot band intensities from main and supplementary data
Supplementary file3 (TIF 25529 KB)

Figures S4. Quantitative analysis of Western blot band intensities from main and supplementary data
Supplementary file4 (TIF 25533 KB)

Figures S5. Quantitative analysis of Western blot band intensities from main and supplementary data
Supplementary file5 (TIF 25533 KB)

Figures S6. Quantitative analysis of Western blot band intensities from main and supplementary data
Supplementary file6 (TIF 25535 KB)

Figures S7. Quantitative analysis of Western blot band intensities from main and supplementary data
Supplementary file7 (TIF 25530 KB)

Figures S8. Quantitative analysis of Western blot band intensities from main and supplementary data
Supplementary file8 (TIF 25531 KB)

Figures S9. Quantitative analysis of Western blot band intensities from main and supplementary data
Supplementary file9 (TIF 25530 KB)

Figures S10. Quantitative analysis of Western blot band intensities from main and supplementary data
Supplementary file10 (TIF 25981 KB)

Figures S11. Quantitative analysis of Western blot band intensities from main and supplementary data
Supplementary file11 (TIF 25531 KB)

Figures S12. Quantitative analysis of Western blot band intensities from main and supplementary data
Supplementary file12 (TIF 25532 KB)

Figures S13. Quantitative analysis of Western blot band intensities from main and supplementary data
Supplementary file13 (TIF 25529 KB)

## Data Availability

No datasets were generated or analysed during the current study.
