# Supplementary figures and images for: Neuroprotective Effects of STAT3 Inhibitor on Hydrogen Peroxide-Induced Neuronal Cell Death via the ERK/CREB Signaling Pathway
Source: Neurochem Res. 2024 Dec 9;50(1):52. doi: 10.1007/s11064-024-04252-3 (PMC11625690; doi:10.1007/s11064-024-04252-3)

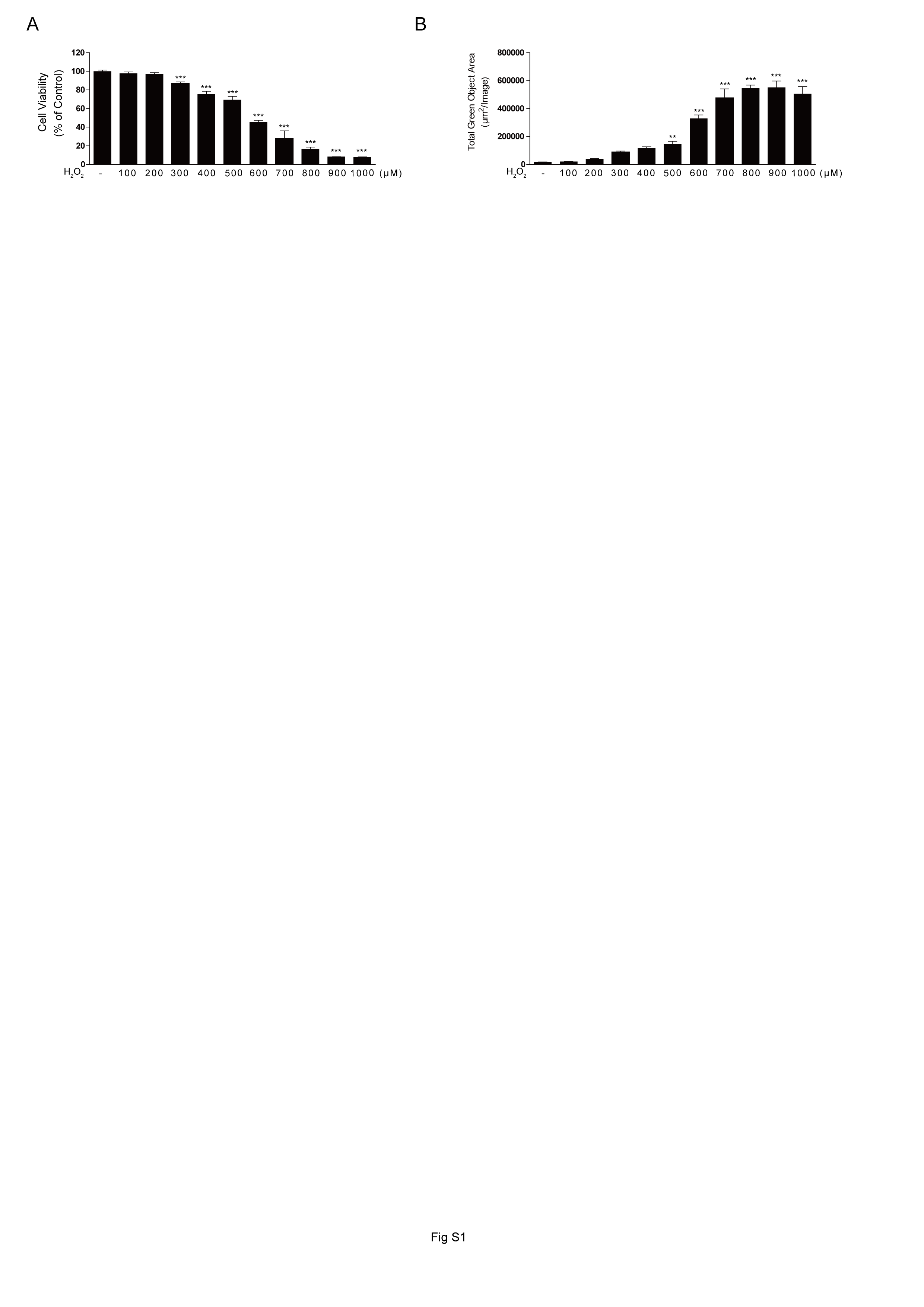

Supplement: Supplementary file 1 — Figure S1. Determination of the optimal H2O2 concentration for inducing oxidative stress in SH-SY5Y cells. SH-SY5Y cells were treated with increasing concentrations of H2O2 (100–1000 μM) for 24 h. (A) Cell viability was assessed using the MTT assay. (B) Cell death assessment was conducted through the quantification of deceased cells using the IncuCyte CytoTox Green Reagent. Supplementary file1 (TIF 25520 KB) [file 11064_2024_4252_MOESM1_ESM.tif]

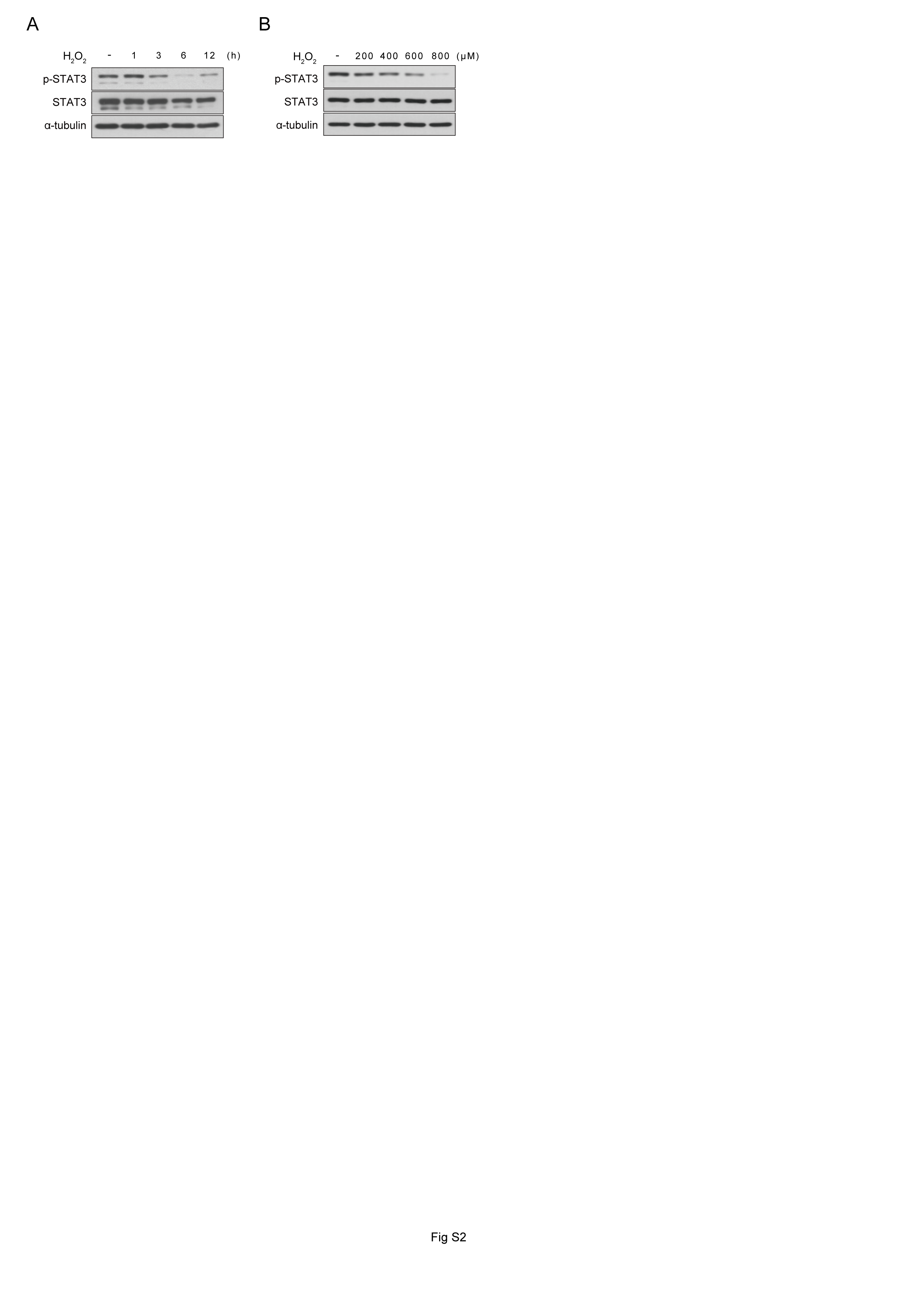

Supplement: Supplementary file 2 — Figure S2. Effects of H2O2 on STAT3 phosphorylation in SH-SY5Y cells. (A) SH-SY5Y cells were treated with H2O2 (600 µM) for various time points. (B) SH-SY5Y cells were treated with different concentrations of H2O2 for 8 h. Western blotting of cell lysates was conducted using the indicated antibodies, and α-tubulin was utilized as a loading control. The data are presented as the mean ± SD, representing a minimum of three independent experiments, with representative data shown. *p < 0.05, **p < 0.01, ***p < 0.005 compared with the control group. All statistical analyses were performed using one-way ANOVA followed by Tukey’s post-hoc test. Supplementary file2 (TIF 25518 KB) [file 11064_2024_4252_MOESM2_ESM.tif]

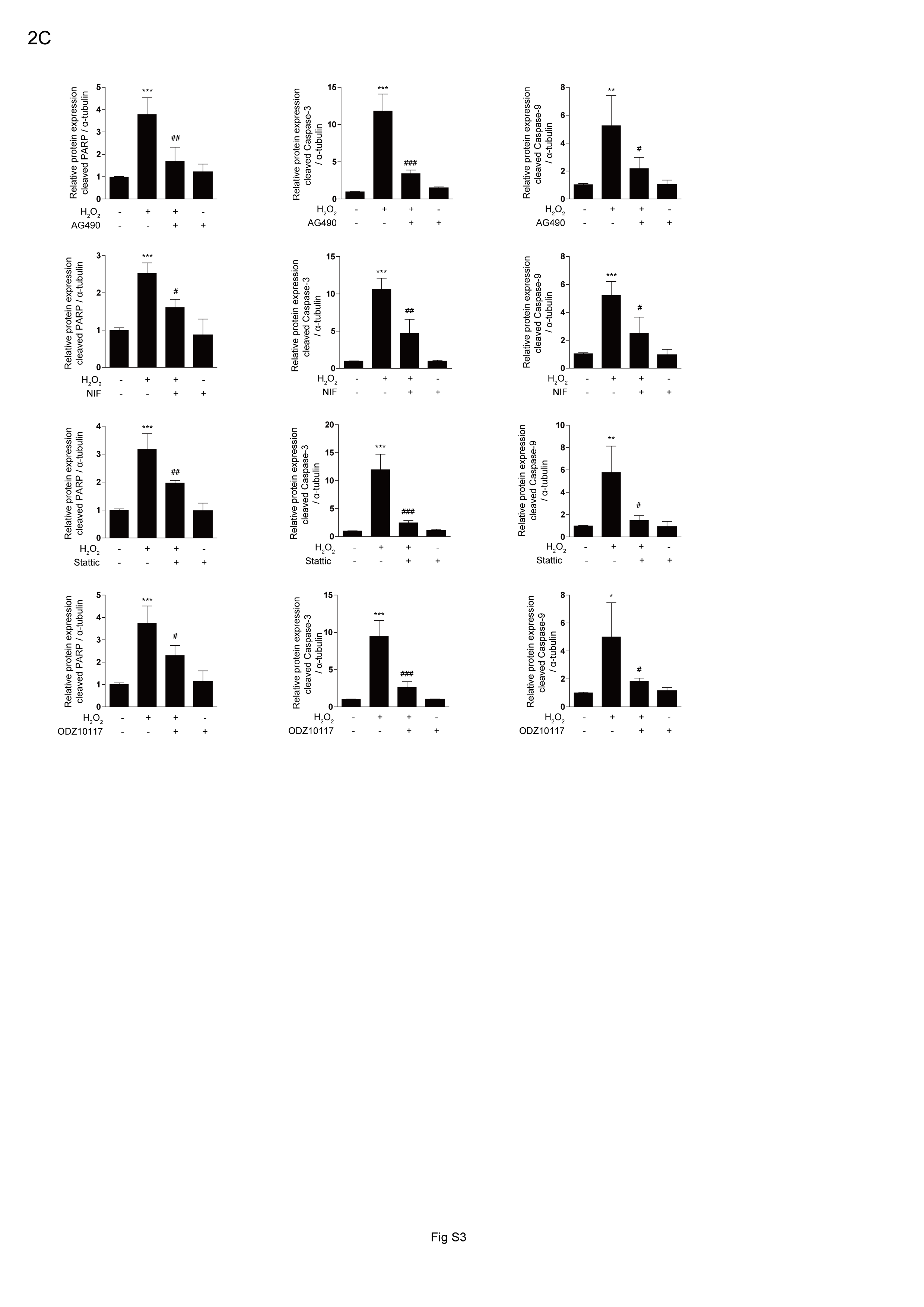

Supplement: Supplementary file 3 — Figures S3–S13. Quantitative analysis of Western blot band intensities from main and supplementary data. The band intensities from the Western blot analyses, including those from the main figures and supplementary materials, were quantified using ImageJ software and normalized to their respective controls. Relative protein expression levels are presented as bar graphs in each figure. The specific normalization controls for each graph are indicated within the corresponding figure. The data are presented as the mean ± SD, representing a minimum of three independent experiments, with representative data shown. NS: not significant, *p < 0.05, **p < 0.01, ***p < 0.005 compared with the control group. #p < 0.05, ##p < 0.01, ###p < 0.005 compared with H2O2-treated group. †p < 0.05, ††p < 0.01, ††† p < 0.005 compared with H2O2+ STAT3 inhibitor-treated group. All statistical analyses were performed using one-way ANOVA followed by Tukey’s post-hoc test. Figures S3. Quantitative analysis of Western blot band intensities from main and supplementary data Supplementary file3 (TIF 25529 KB) [file 11064_2024_4252_MOESM3_ESM.tif]

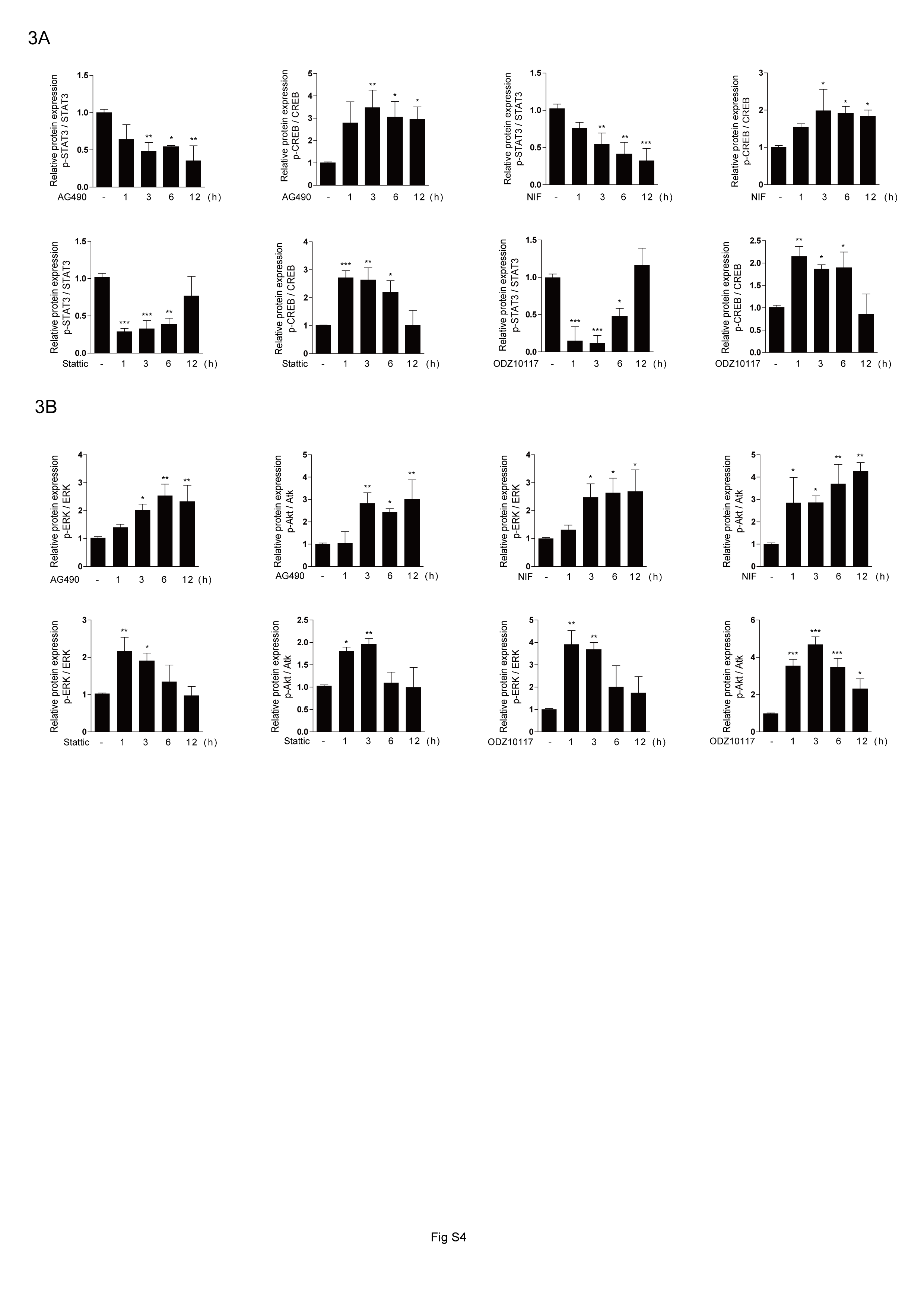

Supplement: Supplementary file 4 — Figures S4. Quantitative analysis of Western blot band intensities from main and supplementary data Supplementary file4 (TIF 25533 KB) [file 11064_2024_4252_MOESM4_ESM.tif]

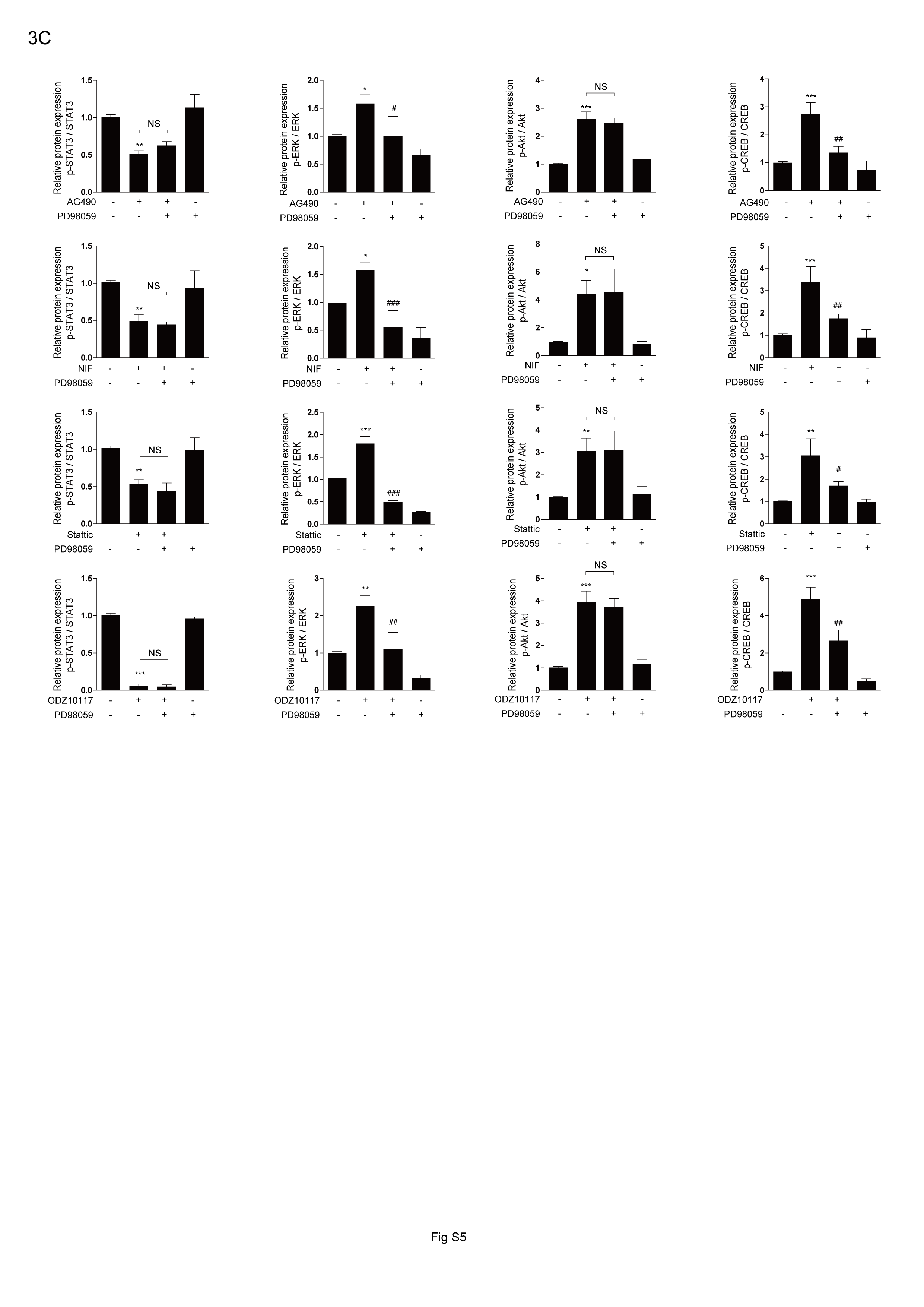

Supplement: Supplementary file 5 — Figures S5. Quantitative analysis of Western blot band intensities from main and supplementary data Supplementary file5 (TIF 25533 KB) [file 11064_2024_4252_MOESM5_ESM.tif]

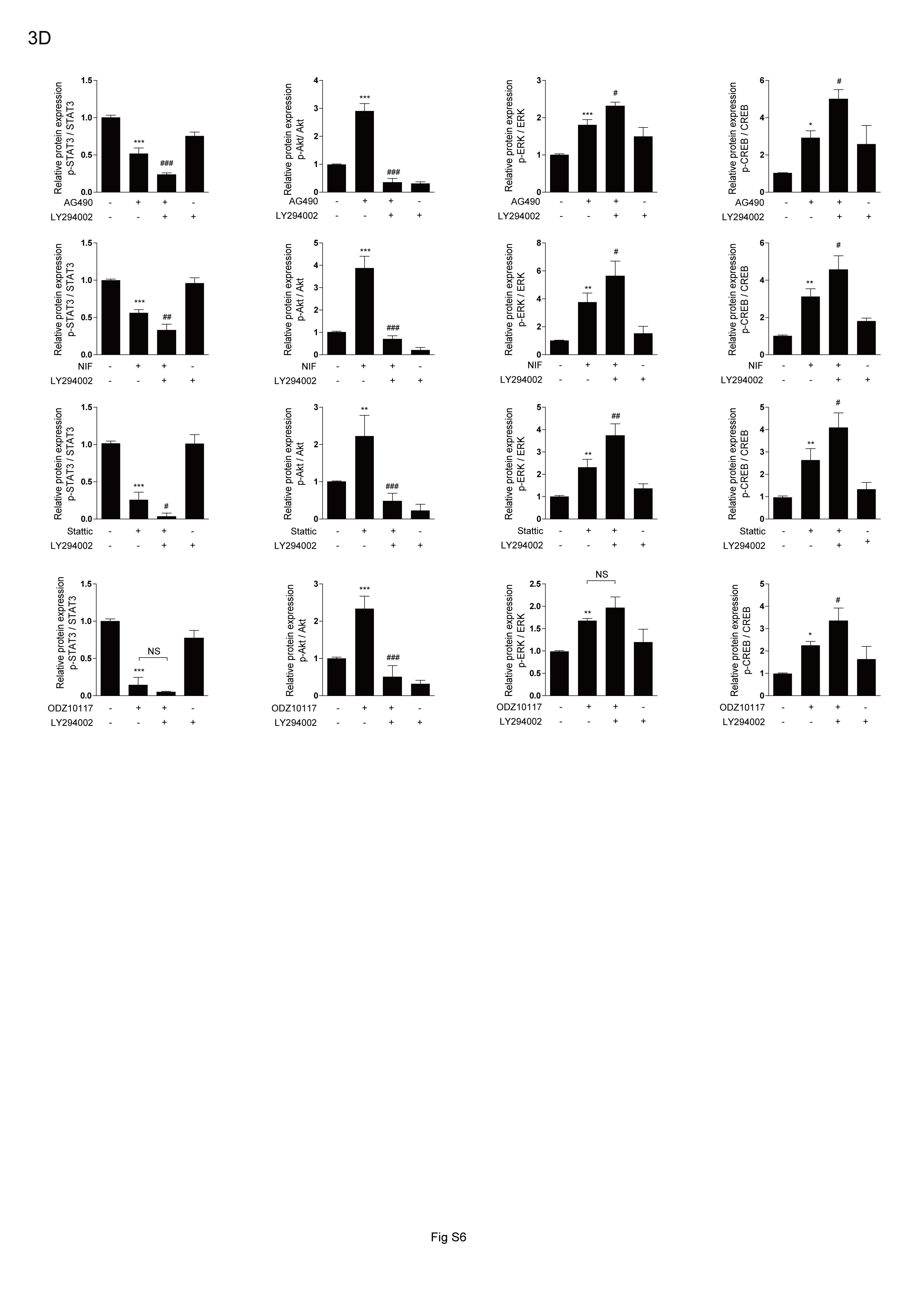

Supplement: Supplementary file 6 — Figures S6. Quantitative analysis of Western blot band intensities from main and supplementary data Supplementary file6 (TIF 25535 KB) [file 11064_2024_4252_MOESM6_ESM.tif]

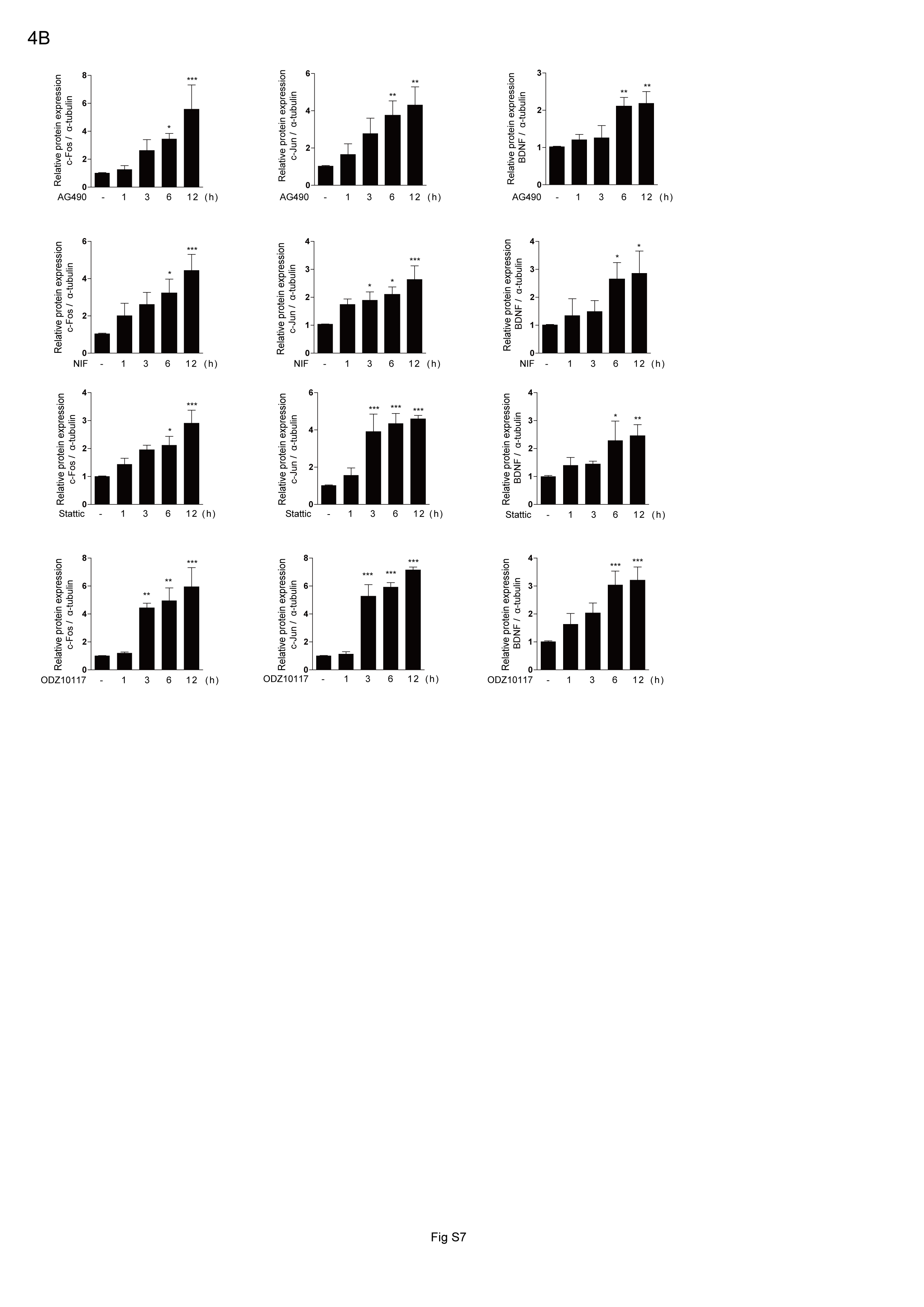

Supplement: Supplementary file 7 — Figures S7. Quantitative analysis of Western blot band intensities from main and supplementary data Supplementary file7 (TIF 25530 KB) [file 11064_2024_4252_MOESM7_ESM.tif]

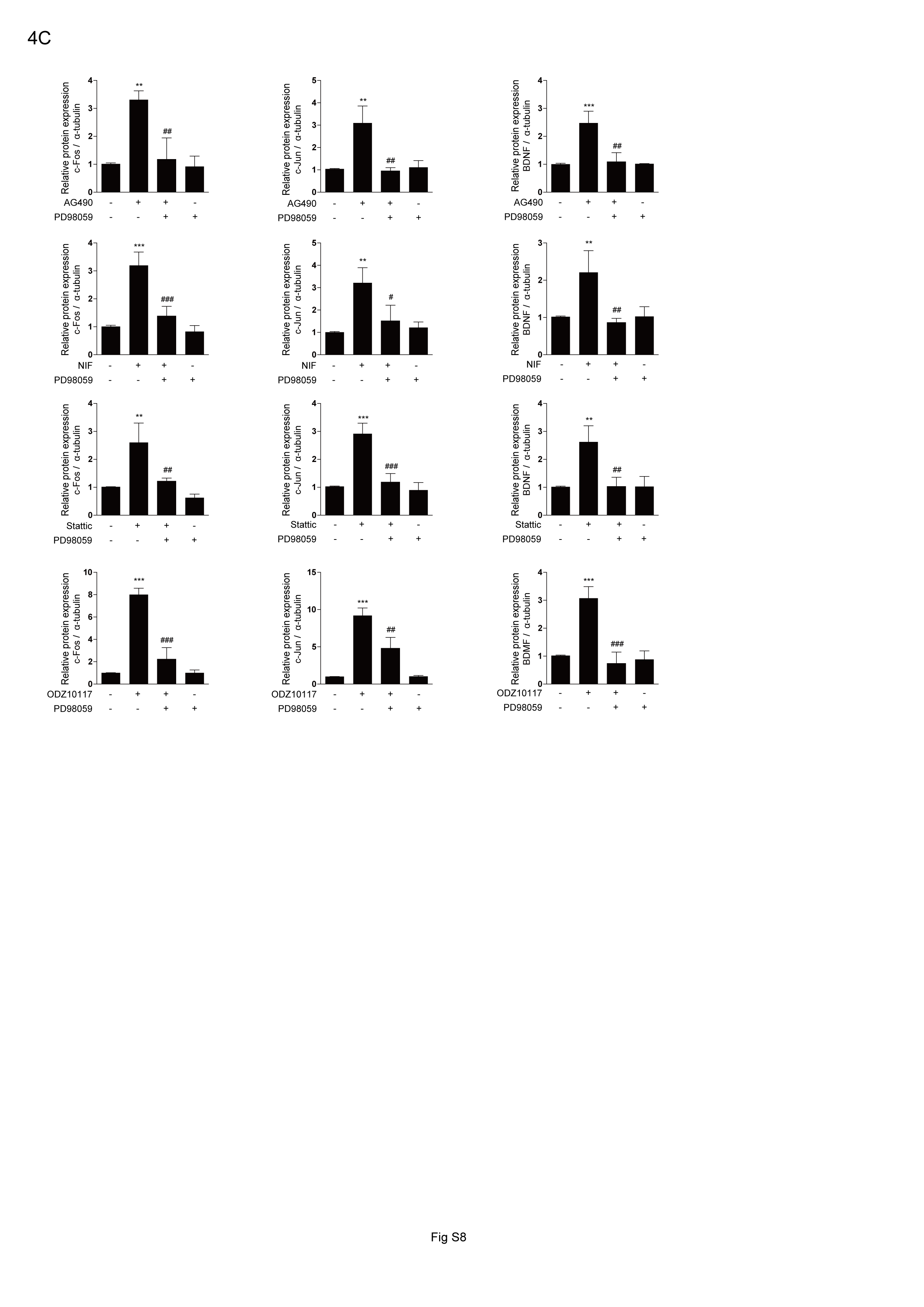

Supplement: Supplementary file 8 — Figures S8. Quantitative analysis of Western blot band intensities from main and supplementary data Supplementary file8 (TIF 25531 KB) [file 11064_2024_4252_MOESM8_ESM.tif]

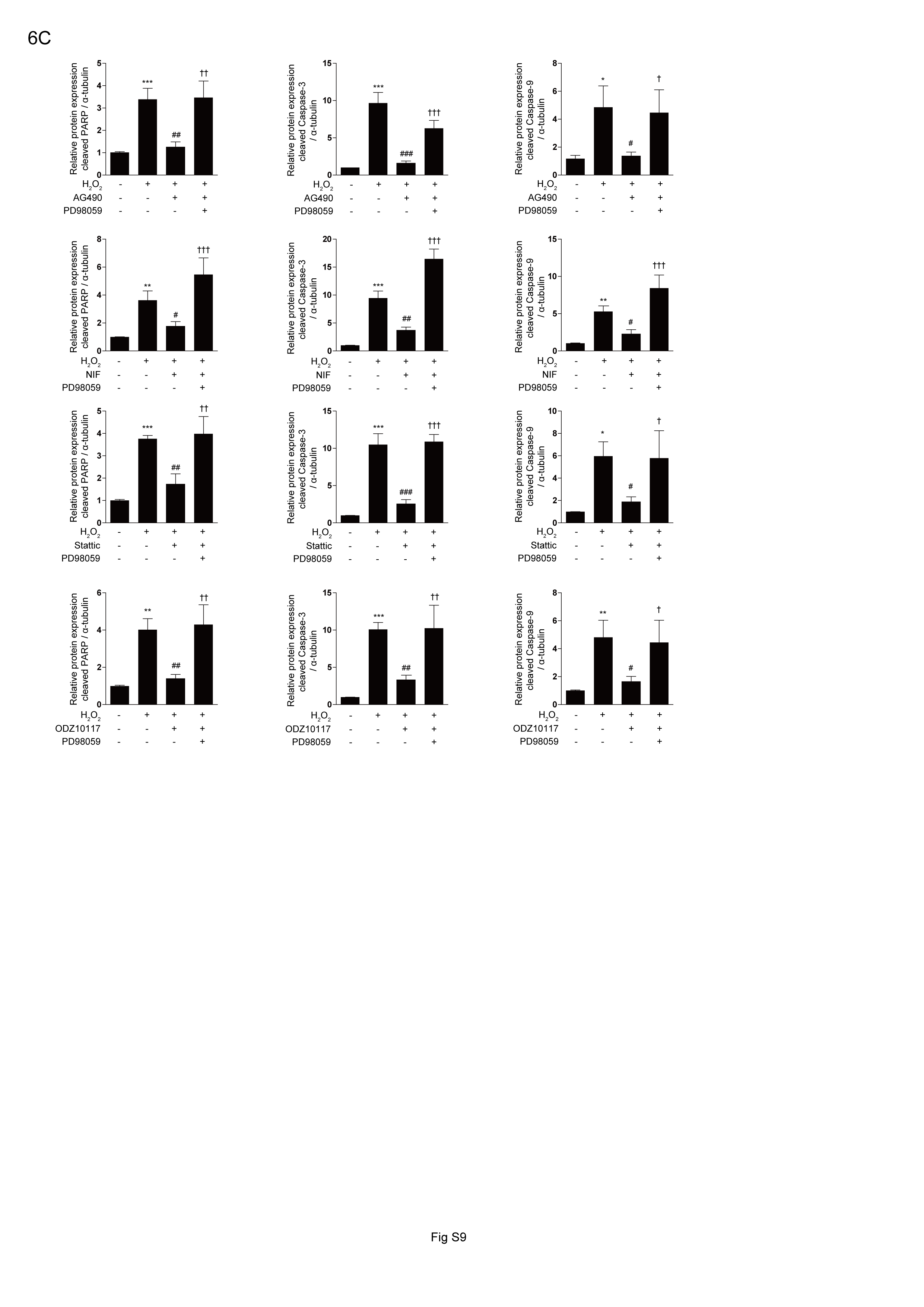

Supplement: Supplementary file 9 — Figures S9. Quantitative analysis of Western blot band intensities from main and supplementary data Supplementary file9 (TIF 25530 KB) [file 11064_2024_4252_MOESM9_ESM.tif]

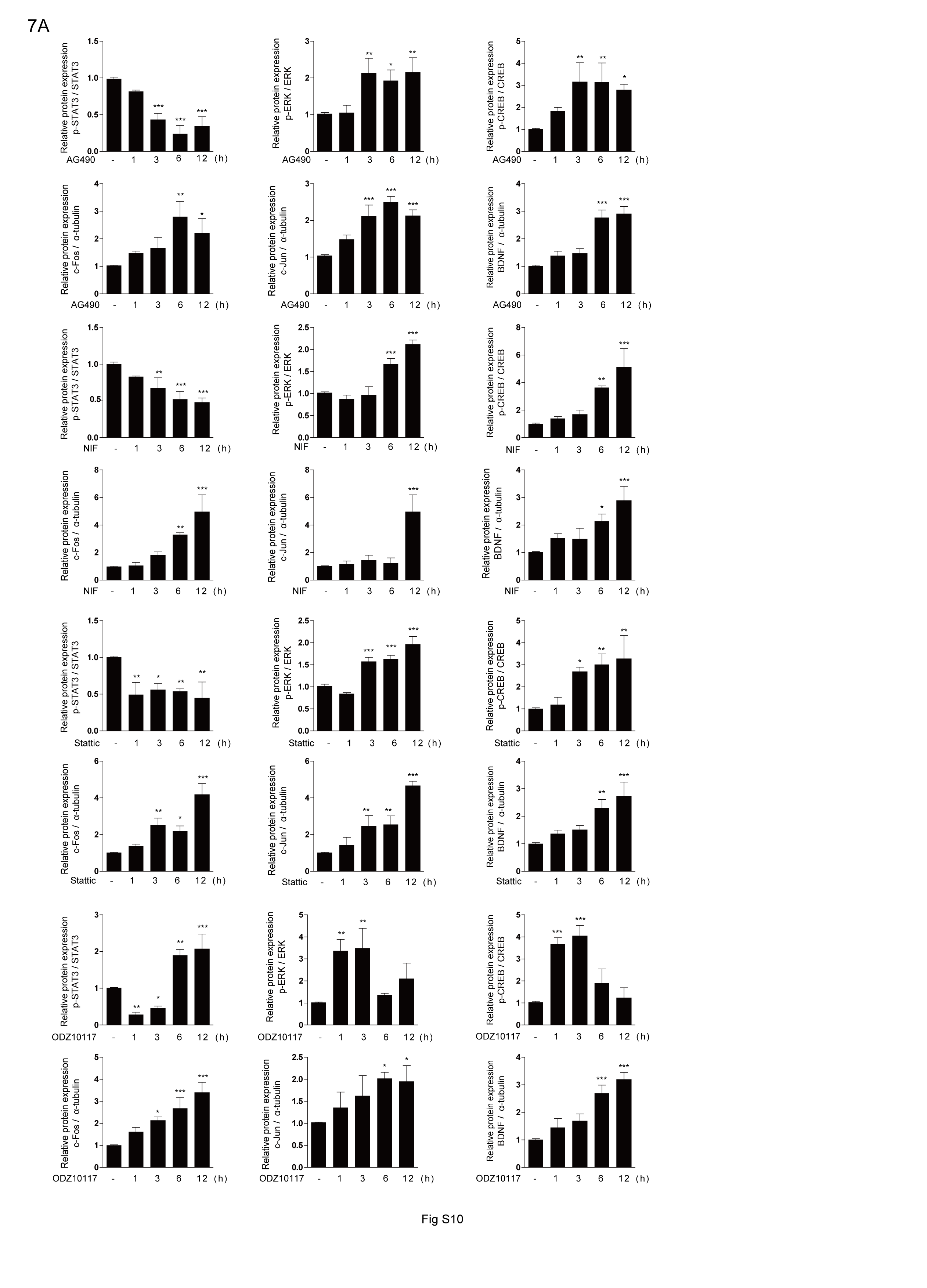

Supplement: Supplementary file 10 — Figures S10. Quantitative analysis of Western blot band intensities from main and supplementary data Supplementary file10 (TIF 25981 KB) [file 11064_2024_4252_MOESM10_ESM.tif]

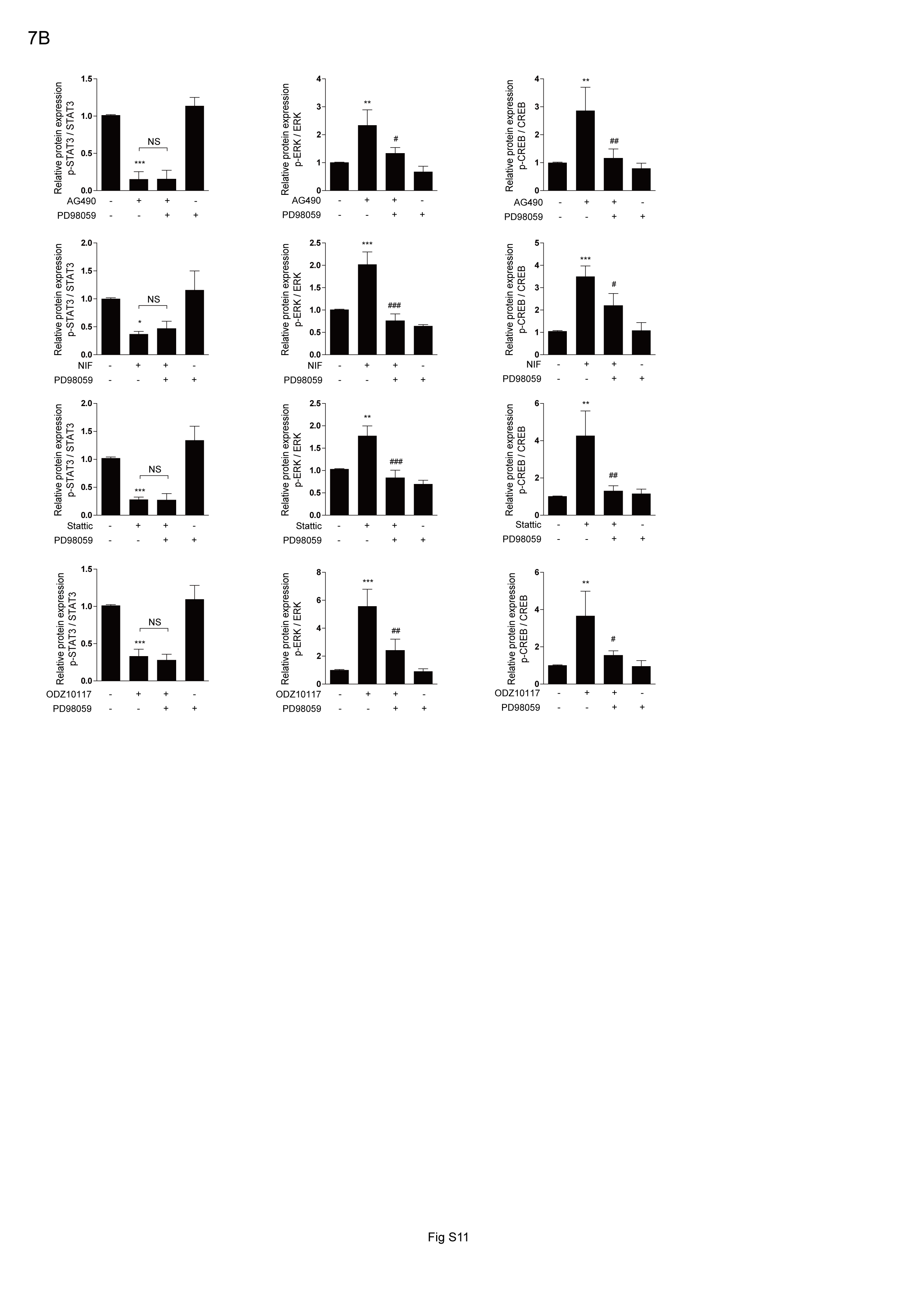

Supplement: Supplementary file 11 — Figures S11. Quantitative analysis of Western blot band intensities from main and supplementary data Supplementary file11 (TIF 25531 KB) [file 11064_2024_4252_MOESM11_ESM.tif]

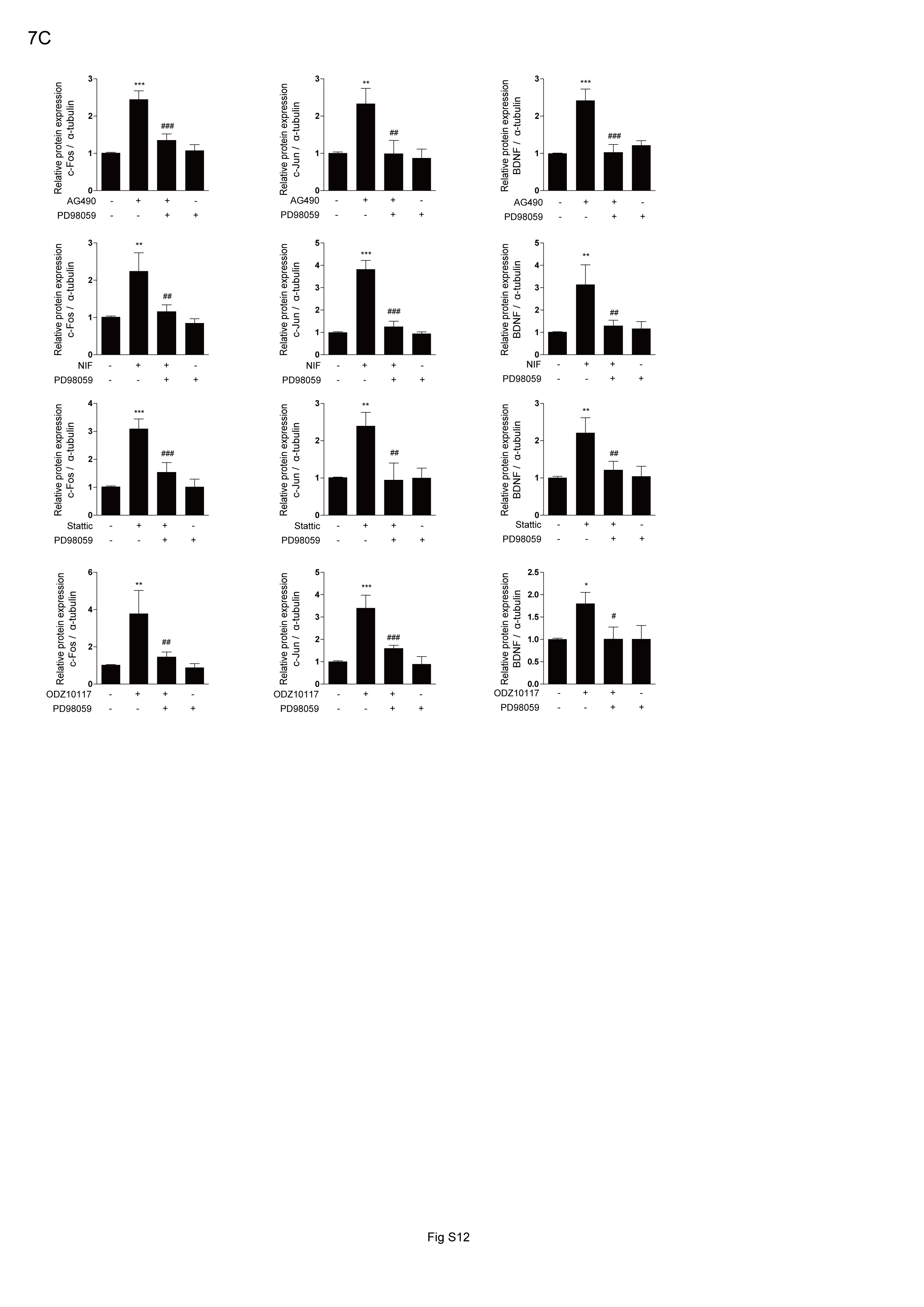

Supplement: Supplementary file 12 — Figures S12. Quantitative analysis of Western blot band intensities from main and supplementary data Supplementary file12 (TIF 25532 KB) [file 11064_2024_4252_MOESM12_ESM.tif]

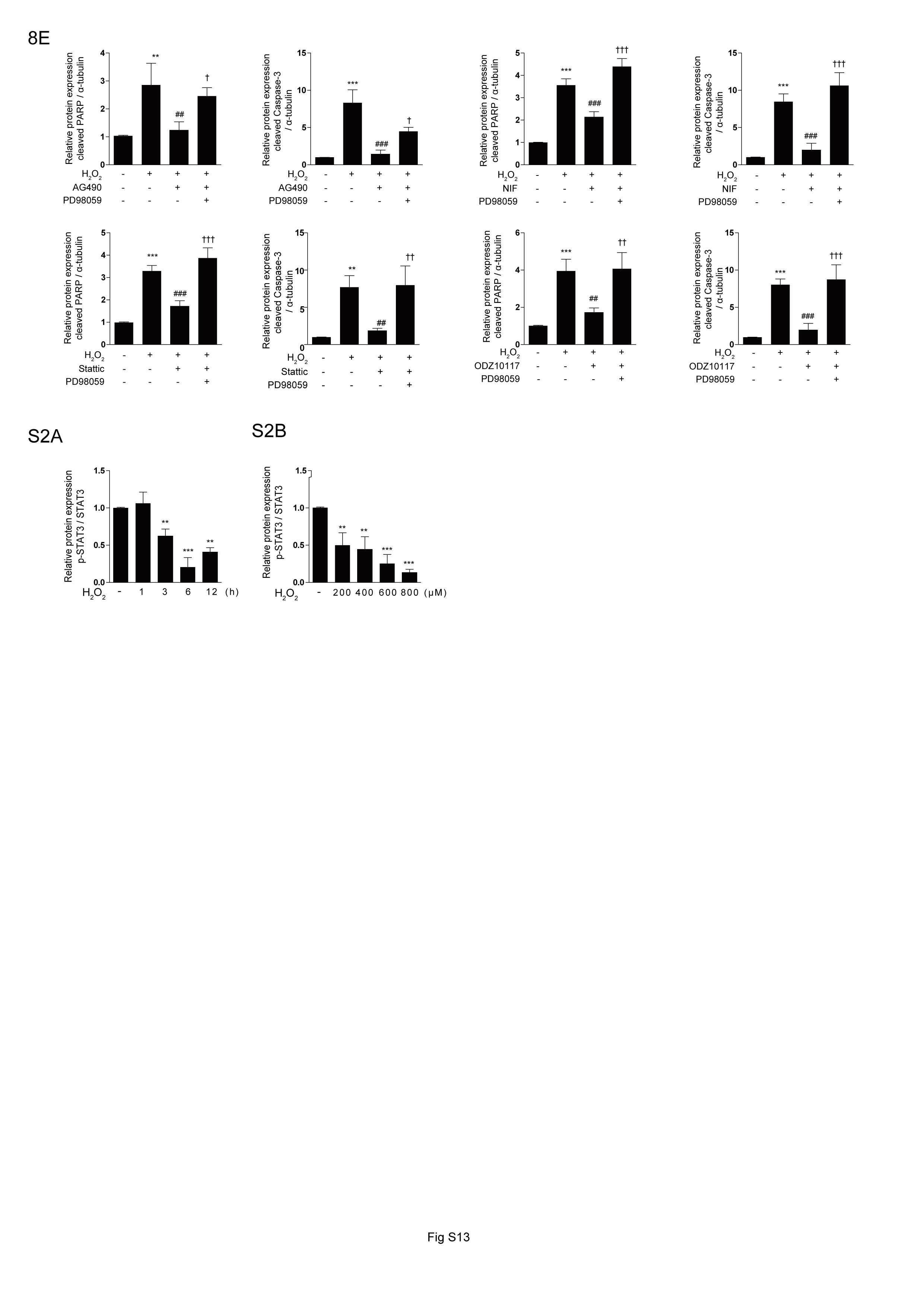

Supplement: Supplementary file 13 — Figures S13. Quantitative analysis of Western blot band intensities from main and supplementary data Supplementary file13 (TIF 25529 KB) [file 11064_2024_4252_MOESM13_ESM.tif]
